# Chaetomugilins and Chaetoviridins—Promising Natural Metabolites: Structures, Separation, Characterization, Biosynthesis, Bioactivities, Molecular Docking, and Molecular Dynamics

**DOI:** 10.3390/jof8020127

**Published:** 2022-01-27

**Authors:** Abdelsattar M. Omar, Gamal A. Mohamed, Sabrin R. M. Ibrahim

**Affiliations:** 1 Department of Pharmaceutical Chemistry, Faculty of Pharmacy, King Abdulaziz University, Jeddah 21589, Saudi Arabia; 2 Center for Artificial Intelligence in Precision Medicines, King Abdulaziz University, Jeddah 21589, Saudi Arabia; 3 Department of Natural Products and Alternative Medicine, Faculty of Pharmacy, King Abdulaziz University, Jeddah 21589, Saudi Arabia; gahussein@kau.edu.sa; 4 Department of Chemistry, Preparatory Year Program, Batterjee Medical College, Jeddah 21442, Saudi Arabia; 5 Department of Pharmacognosy, Faculty of Pharmacy, Assiut University, Assiut 71526, Egypt

**Keywords:** chaetomugilins, chaetoviridins, fungi, Chaetomaceae, characterization, bioactivities, molecular docking, COVID-19, protease, biosynthesis

## Abstract

Fungi are recognized as luxuriant metabolic artists that generate propitious biometabolites. Historically, fungal metabolites have largely been investigated as leads for various therapeutic agents. Chaetomugilins and the closely related chaetoviridins are fungal metabolites, and each has an oxygenated bicyclic pyranoquinone core. They are mainly produced by various Chaetomaceae species. These metabolites display unique chemical features and diversified bioactivities. The current review gives an overview of research about fungal chaetomugilins and chaetoviridins regarding their structures, separation, characterization, biosynthesis, and bioactivities. Additionally, their antiviral potential towards the SARS-CoV-2 protease was evaluated using docking studies and molecular dynamics (MD) simulations. We report on the docking and predictive binding energy estimations using reported crystal structures of the main protease (PDB ID: 6M2N, 6W81, and 7K0f) at variable resolutions—i.e., 2.20, 1.55, and 1.65 Å, respectively. Chaetovirdin D (**43**) exhibited highly negative docking scores of −7.944, −8.141, and −6.615 kcal/mol, when complexed with 6M2N, 6W81, and 7K0f, respectively. The reference inhibitors exhibited the following scores: −5.377, −6.995, and −8.159 kcal/mol, when complexed with 6M2N, 6W81, and 7K0f, respectively. By using molecular dynamics simulations, chaetovirdin D’s stability in complexes with the viral protease was analyzed, and it was found to be stable over the course of 100 ns.

## 1. Introduction

**Figure 1:**
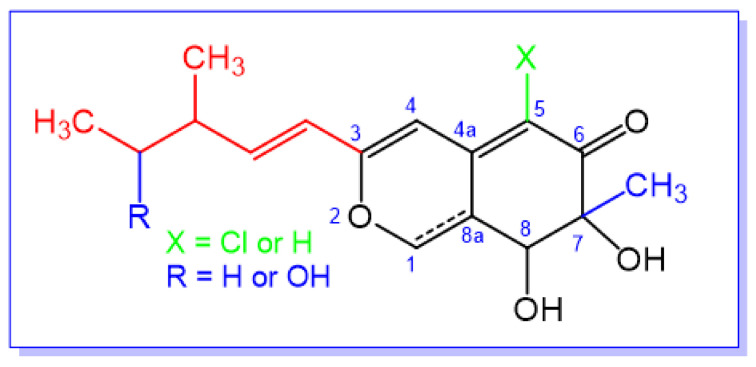
The basic skeleton of chaetomugilins and chaetoviridins.

Fungi are a wealthy and substantial pool of many secondary metabolites with many different structures and diversified bioactivities [1,2,3,4,5,6,7,8,9,10,11]. These metabolites attract much attention as lead metabolites for pharmaceutical agents, and for plant protection [1,8,9,10,11,12,13,14,15,16]. Fungal polyketides (FPKs) represent one of the largest and most structurally diverse groups of fungal metabolites. They range from simple and aromatic to highly macrocyclic and complex [10,13,17]. Their backbone is biosynthesized by the condensation of acyl-CoA thioesters [18]. Their structural variations originate from differences in the starting and extending units, methylation pattern, chain length, degree of reduction, and modifications by tailoring enzymes [19]. Mycotoxins and pigments are among the FPKs that have had remarkable contributions in the field of drug discovery [20]. Azaphilones (azaphilonoids or isochromenes) are fungal pigments belonging to FPKs. Structurally, they have an isochromene skeleton that contains an oxygenated bicyclic pyrano-quinone core and a quaternary carbon center [21]. Biosynthetically, the O atom in the pyran chromophore could be exchanged by an N atom in the existence of primary amines, and accordingly, the pigment color will shift to red [22]. They are produced by various basidiomycetous and ascomycetous fungi, including *Chaetomium*, *Penicillium*, *Aspergillus*, *Talaromyces*, *Phomopsis*, *Monascus*, *Emericella*, *Epicoccum*, *Hypoxylon*, and *Pestalotiopsis*, where they are accountable for the green, red, or yellow color of mycelia and/or fruiting bodies [23]. They possess myriad bioactivities: antitumor, cytotoxic, antimicrobial, anti-inflammatory, antioxidant, enzyme inhibitory, antiviral, insecticidal, and antileishmanial [24]. Chaetomugilins and closely related chaetoviridins are azaphilones featuring a C-7-methyl group and C-5 chlorine—except for chaetomugilins T (**29**) and U (**30**)—and a C-3-branched pentenyl chain (Figure 1). However, chaetomugiline P (**24**) differs from the others in that it has no substituent at C-7 and a methyl group at C-5. 3-Methyl-4-hydroxy-1-pentyl chains at C-3 are found in some chaetomugilins and chaetoviridins. Sometimes, they bear a five-membered lactone and/or a fused tetrahydrofuran/*δ*-lactone [25]. The 7-OH group can have (S) or (R) configuration, though (7S) isomers are the most common among these metabolites. On the other hand, the 7-hydroxyl can be part of a furanone ring [26]. These fungal metabolites are produced by various *Chaetomium* species. Chaetoviridins were firstly reported by Takahashi et al. from *Chaetomium globosum* var. *flavoviride* [27]. Chaetomugilins are known as cytotoxic metabolites, whereas chaetoviridins have antifungal and antibiotic activities [28]. Recently, these metabolites have been recognized as a unique family of fungal metabolites in view of their interesting structural features and prominent bioactivities, which could provoke enormous attention from natural products chemists and pharmacologists. The current review focuses on chaetomugilins and chaetoviridins from fungal sources, including isolation, structural characterization, biosynthesis, and bioactivities (Table 1 and Table 2). Some of the metabolites have been reported with the same names, despite having different structures and molecular formulae—e.g., chaetomugilin S, chaetoviridin B, and chaetoviridin G. Moreover, the structures of some compounds have been revised and renamed: in such cases, both structures have been drawn in the figures, highlighting the new names and corresponding references. Additionally, the emergence of the COVID-19 pandemic motivated us to investigate the potential of these metabolites as antiviral agents towards SARS-CoV-2 using docking studies and molecular dynamics (MD) simulations. Literature searching was carried out using diverse databases—Web of Science, PubMed (MedLine), GoogleScholar, Scopus, and SciFinder—and different publishers— Springer-Link (Cham, Switzerland), Wiley (New York, NY, USA), Taylor & Francis (London, England), Bentham (Sharjah, United Arab Emirates), and ACS (Washington, DC, United States) Publications.

## 2. Extraction, Isolation, and Structural Characterization

For isolation of these metabolites, the fungal materials were extracted with EtOAc or MeOH. Their presence in the extract can be detected by TLC using the solvent systems: EtOAc:toluene:AcOH:formic acid 2.5:7.5:1:1, CHCl_3_:MeOH:AcOH 95:5:0.1, or petroleum spirit/EtOAc 40:60. Upon development, the spots on the SiO_2_ TLC plates can be visualized as dark spots under ultraviolet light (365 nm and 254 nm), or by using ammonia (red color) or ceric ammonium molybdate stain (dark spots; H_2_O (90 mL), H_2_SO_4_ (10 mL), ammonium molybdate (2.5 g), cerium (IV) sulfate (1.0 g)) [27,29,30,31]. Moreover, they gave positive Beilstein reactions [27]. The EtOAc or MeOH extract can be separated by column chromatography using SiO_2_ CC (PE:acetone 9:1–6.5:3.5 or 40:1–2:1; CH_2_Cl_2_:MeOH; cyclohexane:MeOH; EtOAc:CH_2_Cl_2_; cyclohexane:EtOAc; CHCl_3_:MeOH gradient), Sephadex LH-20 (MeOH; CH_2_Cl_2_:MeOH 6:4; CH_2_Cl_2_:MeOH 1:1; CHCl_3_:MeOH 1:1), or MPLC (C-18 ODS) (MeOH:H_2_O gradient) [25,31,32,33]. Finally, the fractions or isolated compounds can be further purified by preparative HPLC (MeOH:H_2_O 90%, 85%, 75%, 65%, or 50% *v*/*v*), CH_3_CN/H_2_O (60:40, 65:35, or 75:25, *v*/*v*) [31,32,33,34,35], or normal phase HPLC (n-hexane:EtOAc 7:3, 4:1, 1:4, 2:1, 1: 3, or 3:2) [36]. Their isolation can also be achieved by loading the sample onto TLC SiO_2_ plates in the form of uniform bands.

**Table 1:** List of chaetomugilins and chaetoviridins. (Molecular weight and formula, fungal source, host, and place of discovery).

Compound Name	Mol. Wt.	Mol. Formula	Fungal Source	Host (Part, Family)	Place	Refs.
Chaetomugilin 106B-6 XXVIII (**1**)	328	C_16_H_21_ClO_5_	*C. globosum*	*Mugii cephalus* (Fish bora stomach content, Mugilidae)	Katsuura, Nachi, Wakayama, Japan	[37]
Chaetomugilin A (**2**)	450	C_23_H_27_ClO_7_	*C. globosum* OUPS-T106B-6	*Mugil cephalus* (Marine fish, Mugilidae)	Katsuura Bay, Japan	[38]
			*C. globosum* OUPS-T106B-6	*Mugil cephalus* (Marine fish, Mugilidae)	Katsuura Bay, Japan	[39]
			*C. globosum* OUPS-T106B-6	*Mugil cephalus* (Marine fish, Mugilidae)	Katsuura Bay, Japan	[40]
			*C. globosum* OUPS-T106B-6	*Mugil cephalus* (Marine fish, Mugilidae)	Katsuura Bay, Japan	[41]
			*C. globosum* OUPS-T106B-6	*Mugil cephalus* (Marine fish, Mugilidae)	Katsuura Bay, Japan	[35]
			*C. globosum*	*Ginkgo biloba* (Leaves, Ginkgoaceae)	Linyi, Shandong, China	[42]
			*C. globosum* OUPS-T106B-6	*Mugil cephalus* (Marine fish, Mugilidae)	Katsuura Bay, Japan	[43]
			*C. globosum* Z1	*Broussonetia papyrifera* (Barks, Moraceae)	Nanjing, Jiangsu, China	[44]
			*C. globosum* TY1	*Ginkgo biloba* (Barks Ginkgoaceae)	Linyi, Shandong, China	[45]
			*C. globosum* CBS148.51	Cultured	China	[46]
			*C. globosum* HDN151398	Sediment sea	South China Sea	[22]
			*C. globosum* TY-2	*Polygonatum sibiricum*, (Root, Convallariaceae)	Linan, Zhejiang, China	[32]
			*C. globosum* TY1	*Ginkgo biloba* (Barks Ginkgoaceae)	Linyi, Shandong, China	[47]
*Seco*-chaetomugilin A (**3**)	482	C_24_H_31_ClO_8_	*C. globosum* OUPS-T106B-6	*Mugil cephalus* (Marine fish, Mugilidae)	Katsuura Bay, Japan	[48]
11-*Epi*-chaetomugilin A (**4**)	450	C_23_H_27_ClO_7_	*C. globosum*	*Mugii cephalus* (Fish bora stomach content, Mugilidae)	Katsuura, Nachi, Wakayama, Japan	[37]
			*C. globosum* OUPS-T106B-6	*Mugil cephalus* (Marine fish, Mugilidae)	Katsuura Bay, Japan	[35]
			*C. globosum* TW1-1	*Armadillidium vulgare* (Pillbugs, Armadillidiidae)	Tongji Medical College, Hubei, China	[33]
			*C. globosum* CBS148.51	Culture	China	[46]
4′-*Epi*-chaetomugilin A (**5**)	450	C_23_H_27_ClO_7_	*C. globosum*	*Mugii cephalus* (Fish bora stomach content, Mugilidae)	Katsuura, Nachi, Wakayama, Japan	[37]
			*C. globosum* OUPS-T106B-6	*Mugil cephalus* (Marine fish, Mugilidae)	Katsuura Bay, Japan	[35]
Chaetomugilin B (**6**)	464	C_24_H_29_ClO_7_	*C. globosum* OUPS-T106B-6	*Mugil cephalus* (Marine fish, Mugilidae)	Katsuura Bay, Japan	[38]
			*C. globosum* OUPS-T106B-6	*Mugil cephalus* (Marine fish, Mugilidae)	Katsuura Bay, Japan	[39]
			*C. globosum* OUPS-T106B-6	*Mugil cephalus* (Marine fish, Mugilidae)	Katsuura Bay, Japan	[40]
			*C. globosum* Z1	*Broussonetia papyrifera* (Barks, Moraceae)	Nanjing, Jiangsu, China	[44]
Chaetomugilin C (**7**)	432	C_23_H_25_ClO_6_	*C. globosum* OUPS-T106B-6	*Mugil cephalus* (Marine fish, Mugilidae)	Katsuura Bay, Japan	[38]
			*C. globosum* OUPS-T106B-6	*Mugil cephalus* (Marine fish, Mugilidae)	Katsuura Bay, Japan	[39]
			*C. globosum* OUPS-T106B-6	*Mugil cephalus* (Marine fish, Mugilidae)	Katsuura Bay, Japan	[40]
			*C. globosum* HDN151398	Sediment sea	South China Sea	[22]
Chaetomugilin D (**8**)	434	C_23_H_27_ClO_6_	*C. globosum*	*Adiantum capillus-veneris* (Plant, Pteridaceae)	Saint Katherine Protectorate, Sinai, Egypt	[49]
			*C. globosum* OUPS-T106B-6	*Mugil cephalus* (Marine fish, Mugilidae)	Katsuura Bay, Japan	[39]
			*C. globosum* OUPS-T106B-6	*Mugil cephalus* (Marine fish, Mugilidae)	Katsuura Bay, Japan	[40]
			*C. globosum*	*Ginkgo bilob* (Leaves, Ginkgoaceae)	Linyi, Shandong province, China	[42]
			*C. globosum* OUPS-T106B-6	*Mugil cephalus* (Marine fish, Mugilidae)	Katsuura Bay, Japan	[41]
			*C. globosum* OUPS-T106B-6	*Mugil cephalus* (Marine fish, Mugilidae)	Katsuura Bay, Japan	[43]
			*C. globosum* DAOM 240359	Indoor air samples or building materials	Ontario, Alberta, Saskatchewan, Nova Scotia, Canada	[30]
			*C. globosum* DAOM 240359	Indoor air samples or building materials	Ontario, Alberta, Saskatchewan, Nova Scotia, Canada	[50]
			*C. globosum*	*Amaranthus viridis* (Leaves, Amaranthaceae)	Central Province of Sri Lanka	[51]
			*C. globosum* TW1-1	*Armadillidium vulgare* (Pill bugs, Armadillidiidae)	Tongji Medical College, Hubei Province, China	[33]
			*C. globosum* DAOMC 240359	Damp building materials	Ontario, Alberta, Saskatchewan, Nova Scotia, Canada	[52]
			*C. globosum* TY1	*Ginkgo biloba* (Barks Ginkgoaceae)	Linyi, Shandong, China	[45]
			*C. globosum* CBS148.51	Cultured	China	[46]
			*Chaetomium* sp. NA-S01-R1	Deep-sea	West Pacific Ocean, China	[25]
			*C. cochliodes*	Indoor buildings	Finland	[53]
			*C. globosum*	Seawater and marine deposits	Jeju, Korea	[54]
			*C. globosum* DAOM 240359	Damp and moldy buildings	Canada	[55]
*Seco*-chaetomugilin D (**9**)	466	C_24_H_31_ClO_7_	*C. globosum* OUPS-T106B-6	*Mugil cephalus* (Marine fish, Mugilidae)	Katsuura Bay, Japan	[48]
			*C. globosum*	*Wikstroemia uva-ursi* (Leaves, Thymelaeaceae)	Hawaiian Islands, USA	[56]
			*C. cupreum*	National Fungal Culture Collection	India, Agharkar Research Institute, Pune, India	[29]
*Epi*-chaetomugilin D (**10**)	434	C_23_H_27_ClO_6_	*C. globosum*	*Adiantum capillus-veneris* (Plant, Pteridaceae)	Saint Katherine Protectorate, Sinai, Egypt	[49]
Chaetomugilin E (**11**)	448	C_24_H_29_ClO_6_	*C. globosum* OUPS-T106B-6	*Mugil cephalus* (Marine fish, Mugilidae)	Katsuura Bay, Japan	[39]
			*C. globosum* OUPS-T106B-6	*Mugil cephalus* (Marine fish, Mugilidae)	Katsuura Bay, Japan	[40]
			*C. globosum*	*Wikstroemia uva-ursi* (Leaves, Thymelaeaceae)	Hawaiian Islands, USA	[56]
Chaetomugilin EA-4 (**12**)	406	C_22_H_27_ClO_5_	*C. globosum* Kunze ex. 5157	Soils of wheat field	New Delhi, India	[57]
Chaetomugilin F (**13**)	416	C_23_H_25_ClO_5_	*C. globosum* OUPS-T106B-6	*Mugil cephalus* (Marine fish, Mugilidae)	Katsuura Bay, Japan	[39]
			*C. globosum* OUPS-T106B-6	*Mugil cephalus* (Marine fish, Mugilidae)	Katsuura Bay, Japan	[40]
			*C. globosum*	*Wikstroemia uva-ursi* (Leaves, Thymelaeaceae)	Hawaiian Islands, USA	[56]
Chaetomugilin G (**14**)	464	C_24_H_29_ClO_7_	*C. globosum* OUPS-T106B-6	*Mugil cephalus* (Marine fish, Mugilidae)	Katsuura Bay, Japan	[39]
			*C. globosum* OUPS-T106B-6	*Mugil cephalus* (Marine fish, Mugilidae)	Katsuura Bay, Japan	[41]
Chaetomugilin H (**15**)	448	C_24_H_29_ClO_6_	*C. globosum* OUPS-T106B-6	*Mugil cephalus* (Marine fish, Mugilidae)	Katsuura Bay, Japan	[39]
			*C. globosum* OUPS-T106B-6	*Mugil cephalus* (Marine fish, Mugilidae)	Katsuura Bay, Japan	[41]
Chaetomugilin I (**16**)	406	C_22_H_27_ClO_5_	*C. globosum*	*Mugii cephalus* (Fish bora stomach content, Mugilidae)	Katsuura, Nachi, Wakayama, Japan	[37]
			*C. globosum*	*Mugil cephalus* (Marine fish, Mugilidae)	Katsuura Bay, Japan	[58]
			*C. globosum*	*Mugil cephalus* (Marine fish, Mugilidae)	Katsuura Bay, Japan	[58]
			*C. globosum*	*Wikstroemia uva-ursi* (Leaves, Thymelaeaceae)	Hawaiian Islands, USA	[56]
			*C. globosum* TY1	*Ginkgo biloba* (Barks Ginkgoaceae)	Linyi, Shandong, China	[45]
			*C. globosum* TY-2	*Polygonatum sibiricum*, (Roots, Convallariaceae)	Linan, Zhejiang, China	[32]
			*C. globosum* TY1	*Ginkgo biloba* (Barks, Ginkgoaceae)	Linyi, Shandong, China	[47]
			*C. globosum* Kunze ex. 5157	Soils of wheat field	New Delhi, India	[57]
11-*Epi*-chaetomugilin I (**17**)	406	C_22_H_27_ClO_5_	*C. globosum* OUPS-T106B-6	*Mugil cephalus* (Marine fish, Mugilidae)	Katsuura Bay, Japan	[59]
			*C. globosum*	*Wikstroemia uva-ursi* (Leaves, Thymelaeaceae)	Hawaiian Islands, USA	[56]
Chaetomugilin J (**18**)	390	C_22_H_27_ClO_4_	*C. globosum*	*Mugil cephalus* (Marine fish, Mugilidae)	Katsuura Bay, Japan	[58]
			*C. globosum*	*Adiantum capillus-veneris* (Plant, Pteridaceae)	Saint Katherine Protectorate, Sinai, Egypt	[49]
			*C. globosum*	*Mugii cephalus* (Fish bora stomach content, Mugilidae)	Katsuura, Nachi, Wakayama, Japan	[37]
			*C. globosum*	*Amaranthus viridis* (Leaves, Amaranthaceae)	Central Province of Sri Lanka	[51]
			*C. globosum*	*Wikstroemia uva-ursi* (Leaves, Thymelaeaceae)	Hawaiian Islands, USA	[56]
			*C. globosum* TY1	*Ginkgo biloba* (Barks, Ginkgoaceae)	Linyi, Shandong, China	[45]
			*C. globosum* TY-2	*Polygonatum sibiricum* (Root, Convallariaceae)	Linan, Zhejiang, China	[32]
			*C. globosum*	*Polygonatum sibiricum*, (Root, Convallariaceae)	China	[34]
			*C. globosum* TY1	*Ginkgo biloba* (Barks, Ginkgoaceae)	Linyi, Shandong, China	[47]
			*C. globosum* Kunze ex. 5157	Soils of wheat field	New Delhi, India	[57]
Chaetomugilin K (**19**)	420	C_23_H_29_ClO_5_	*C. globosum*	*Mugil cephalus* (Marine fish, Mugilidae)	Katsuura Bay, Japan	[58]
Chaetomugilin L (**20**)	404	C_23_H_29_ClO_4_	*C. globosum*	*Mugil cephalus* (Marine fish, Mugilidae)	Katsuura Bay, Japan	[58]
Chaetomugilin M (**21**)	450	C_23_H_27_ClO_7_	*C. globosum*	*Mugil cephalus* (Marine fish, Mugilidae)	Katsuura Bay, Japan	[58]
Chaetomugilin N (**22**)	432	C_23_H_25_ClO_6_	*C. globosum*	*Mugil cephalus* (Marine fish, Mugilidae)	Katsuura Bay, Japan	[58]
			*C. globosum*	*Wikstroemia uva-ursi* (Leaves, Thymelaeaceae)	Hawaiian Islands, USA	[56]
Chaetomugilin O (**23**)	416	C_23_H_25_ClO_5_	*C. globosum*	*Mugil cephalus* (Marine fish, Mugilidae)	Katsuura Bay, Japan	[58]
			*C. globosum* TY1	*Ginkgo biloba* (Barks, Ginkgoaceae)	Linyi, Shandong, China	[45]
			*C. globosum* TY-2	*Polygonatum sibiricum*, (Root, Convallariaceae)	Linan, Zhejiang, China	[32]
Chaetomugilin P (**24**)	406	C_22_H_27_ClO_5_	*C. globosum* OUPS-T106B-6	*Mugil cephalus* (Marine fish, Mugilidae)	Katsuura Bay, Japan	[59]
Chaetomugilin Q (**25**)	424	C_22_H_29_ClO_6_	*C. globosum* OUPS-T106B-6	*Mugil cephalus* (Marine fish, Mugilidae)	Katsuura Bay, Japan	[59]
			*C. globosum* TW1-1	*Armadillidium vulgare* (Pill bugs, Armadillidiidae)	Tongji Medical College, Hubei Province, China	[33]
			*C. globosum* TY1	*Ginkgo biloba* (Barks, Ginkgoaceae)	Linyi, Shandong, China	[45]
			*C. globosum* TY1	*Ginkgo bilob* (Leaves, Ginkgoaceae)	Linyi, Shandong province, China	[47]
			*C. globosum* TY-2	*Polygonatum sibiricum*, (Root, Convallariaceae)	Linan, Zhejiang, China	[32]
Chaetomugilin R (**26**)			*C. globosum* OUPS-T106B-6	*Mugil cephalus* (Marine fish, Mugilidae)	Katsuura Bay, Japan	[59]
Chaetomugilin S (**27**)	434	C_23_H_27_ClO_6_	*C. globosum* OUPS-T106B-6	*Mugil cephalus* (Marine fish, Mugilidae)	Katsuura Bay, Japan	[43]
			*C. globosum* TW1-1	*Armadillidium vulgare* (Pillbugs, Armadillidiidae)	Tongji Medical College, Hubei Province, China	[33]
			*C. globosum* TY1	*Ginkgo biloba* (Barks, Ginkgoaceae)	Linyi, Shandong, China	[45]
Chaetomugilin S (**28**)	420	C_23_H_29_ClO_5_	*C. elatum* No. 89-1-3-1	*Ramalina calicaris* (Lichen, Ramalinaceae)	Zixishan Mountain, Yunnan, China	[31]
Chaetomugilin T (**29**)	416	C_23_H_28_O_7_	*C. globosum* OUPS-T106B-6	*Mugil cephalus* (Marine fish, Mugilidae)	Katsuura Bay, Japan	[43]
Chaetomugilin U (**30**)	406	C_23_H_28_O_6_	*C. globosum* OUPS-T106B-6	*Mugil cephalus* (Marine fish, Mugilidae)	Katsuura Bay, Japan	[43]
Chaetoviridin A (**31**)	432	C_23_H_25_ClO_6_	*C. globosum* DAOM 240359	Indoor air samples or building materials	Ontario, Alberta, Saskatchewan, Nova Scotia, Canada	[30,50]
			*Chaetomium* sp. NA-S01-R1	Deep sea	West Pacific Ocean, China	[25]
			*C. cochliodes* VTh01 *C. cochliodes* CTh05	Soil	Ubon Rajathanee province, Bangkok, Thailand Chiangrai province, Bangkok, Thailand	[60]
			*C. globosum*	*Adiantum capillus-veneris* (Plant, Pteridaceae)	Saint Katherine Protectorate, Sinai, Egypt	[49]
			*C. globosum* 5157	Soils of wheat field	New Delhi, India	[57]
			C. globosum var. *flavo-viride*	Culture	-	[27]
			*C. globosum* F0142	*Echinochloa crusgalli* (Stems, Poaceae)	Korea	[61]
			*C. globosum*	*Viguiera robusta* (Leaves, Asteraceae)	Spain	[36]
			*C. siamense*	Soil	Bangkok, Thailand	[62]
			*C. globosum*	Cucumber soil (Rhizosphere)	Egypt	[63]
			*C. globosum* CIB-160	-	China	[64]
			*C. subafine*	Culture	Japan	[65]
			*C. globosum* DAOM 240359	Damp and moldy buildings	Canada	[55]
			*C. globosum*	Sea water and marine deposits	Jeju, Korea	[54]
			*C. globosum* F211_UMNG	*Protium heptaphyllum* (Leaves, Burseraceae)	Foothill of the west Colombian Andes mountains, Aguazul, Casanare, Colombia	[66]
			*C. globosum* 22-10	Soil	PaLong ZangBu Brook, Tibet, China	[67]
			*C. globosum*	*Artemisia desterorum* (Roots, Asteraceae)	Tengger Desert, Ningxia Province, China	[68]
			*C*. *globosum* CEF-082	*Gossypium arboreum* (Plant, Malvaceae)	China	[69]
			*C. globosum* E-C-2	*Apostichopus japonicas* (Surface muscle, Stichopodidae)	Chengshantou Island, Weihai City, the Yellow Sea, China	[70]
			*C. globosum*	Indoor buildings	Finland	[53]
			*C. cochliodes*	indoor buildings	Finland	[53]
			*C. globosum* MP4-S01-7	Sea water	West Pacific Ocean, China	[71]
4′-*Epi*-chaetoviridin A (**32**)	432	C_23_H_25_ClO_6_	*C. globosum*	*Viguiera robusta* (Leaves, Asteraceae)	Spain	[36]
			*C. globosum* F211_UMNG	*Protium heptaphyllum* (Leaves, Burseraceae)	Foothill of the west Colombian Andes mountains, Aguazul, Casanare, Colombia	[66]
5′-*Epi*-chaetoviridin A (**33**)	432	C_23_H_25_ClO_6_	*C. cochliodes* VTh01 *C. cochliodes* CTh05	Soil	Ubon Rajathanee province, Bangkok, Thailand Chiangrai province, Bangkok, Thailand	[60]
			*C. globosum*	*Viguiera robusta* (Leaves, Asteraceae)	Spain	[36]
			*C. globosum* TY1	*Ginkgo biloba* (Barks, Ginkgoaceae)	Linyi, Shandong province, China	[72]
			*C. globosum* CDW7,	*Ginkgo biloba* (Leaves, Ginkgoaceae)	China	[73]
			*C. globosum* 22-10	Soil	Palong Zangbu Brook, Tibet, China	[67]
			*C. globosum* F211_UMNG	*Protium heptaphyllum* (Leaves, Burseraceae)	Foothill of the west Colombian Andes mountains, Aguazul, Casanare, Colombia	[66]
7,5′-*Bis*-*epi*-chaetoviridin A (**34**)	432	C_23_H_25_ClO_6_	*C. elatum* No. 89-1-3-1	*Ramalina calicaris* (Lichen, Ramalinaceae)	Zixishan Mountain, Yunnan, China	[31]
*N*-Glutarylchaetoviridin A (**35**)	603	C_31_H_38_ClNO_9_	*C. globosum* HDN151398	Sea sediment	South China Sea	[22]
4′-*Epi*-N-2-Hydroxyethyl-azachaetoviridin A (**36**)	475	C_25_H_30_ClNO_6_	*C. globosum* DAOM 240359	Indoor air samples or building materials	Ontario, Alberta, Saskatchewan, Nova Scotia, Canada	[50]
Chaetoviridin B (**37**)	452	C_23_H_29_ClO_7_	*C. globosum* var. *flavo-viride*	Culture	-	[27]
			*C. globosum* F0142	*Echinochloa crusgalli* (Stems, Poaceae)	Korea	[61]
			*C. globosum*	*Viguiera robusta* (Leaves, Asteraceae)	Spain	[36]
			*C. globosum*	Cucumber soil (Rhizosphere)	Egypt	[63]
Chaetoviridin B (**38**)			*C. globosum* 5157	Soils of wheat field	New Delhi, India	[57]
			*C. globosum* E-C-2	*Apostichopus japonicas* (Surface muscle, Stichopodidae)	Chengshantou Island, Weihai City, the Yellow Sea, China	[70]
			*C. globosum* Z1	*Broussonetia papyrifera* (Barks, Moraceae)	Nanjing, Jiangsu, China	[44]
			*C. globosum* TY1	*Ginkgo biloba* (Barks, Ginkgoaceae)	Linyi, Shandong, China	[47]
			*Chaetomium* sp.	*Dromaius novaehollandiae* (Scat, Casuariidae)	Australia	[74]
			*C. globosum*	*Adiantum capillus-veneris* (Plant, Pteridaceae)	Saint Katherine Protectorate, Sinai, Egypt	[49]
			*C. globosum* TY1	*Ginkgo biloba* (Barks, Ginkgoaceae)	Linyi, Shandong province, China	[72]
*N*-Glutarylchaetoviridin B (**39**)	543	C_28_H_30_ClNO_8_	*C. globosum* HDN151398	Sea sediment	South China Sea	[22]
Chaetoviridin C (**40**)	434	C_23_H_27_ClO_6_	*C. globosum* var. *flavo-viride*	Culture	-	[27]
			*C. globosum*	*Viguiera robusta* (Leaves, Asteraceae)	Spain	[36]
			*Chaetomium globosum* OUPS-T106B-6	*Mugil cephalus* (Marine fish, Mugilidae)	Katsuura Bay, Japan	[41]
			*C. globosum*	Indoor buildings	Finland	[53]
12β-Hydroxychaetoviridin C (**41**)	450	C_23_H_27_ClO_7_	*C. globosum*	*Viguiera robusta* (Leaves, Asteraceae)	Spain	[36]
*N*-Glutarylchaetoviridin C (**42**)	571	C_30_H_34_ClNO_8_	*C. globosum* HDN151398	Sea sediment	South China Sea	[22]
Chaetoviridin D (**43**)	486	C_23_H_29_ClO_8_	C. globosum var. *flavo-viride*	Culture	Spain	[27]
			*C. globosum*	*Viguiera robusta* (Leaves, Asteraceae)	Spain	[36]
			*Chaetomium* sp.	*Dromaius novaehollandiae* (Scat, Casuariidae)	Australia	[74]
Chaetoviridin E (**44**)	414	C_23_H_23_ClO_5_	*C. globosum* 5157	Soils of wheat field	New Delhi, India	[57]
			*C. globosum* MP4-S01-7	Sea water	West Pacific Ocean, China	[71]
			*C. globosum*	*Artemisia desterorum* (Roots, Asteraceae)	Tengger Desert in Ningxia, China.	[68]
			*C. globosum* E-C-2	*Apostichopus japonicas* (Surface muscle, Stichopodidae)	Chengshantou Island, Weihai City, the Yellow Sea, China	[70]
			*Chaetomium* sp. NA-S01-R1	Deep sea	West Pacific Ocean, China	[25]
			*C. globosum* 22-10	Soil	PaLong ZangBu Brook, Tibet, China	[67]
			*C. globosum*	Sea water and marine deposits	Jeju, Korea	[54]
			*C. cochliodes* CTh05	Soil	Ubon Rajathanee province, Bangkok, Thailand	[60]
			*C. globosum*	*Adiantum capillus-veneris* (Plant, Pteridaceae)	Saint Katherine Protectorate, Sinai, Egypt	[49]
			*C. globosum*	*Viguiera robusta* (Leaves, Asteraceae)	Spain	[36]
			*Chaetomium siamense*	Soil	Bangkok, Thailand	[62]
			*Chaetomium* sp.	*Dromaius novaehollandiae* (Scat, Casuariidae)	Australia	[74]
			*C. globosum* TY1	*Ginkgo biloba* (Barks, Ginkgoaceae)	Linyi, Shandong province, China	[72]
7-*Epi*-chaetoviridin E (**45**)	414	C_23_H_23_ClO_5_	*C. elatum* No. 89-1-3-1	*Ramalina calicaris* (Lichen, Ramalinaceae)	Zixishan Mountain, Yunnan, China	[31]
N-2-Butyric-azochaetoviridin E (**46**)	499	C_27_H_30_ClNO_6_	*C. globosum* DAOM 240359	Indoor air samples or building materials	Ontario, Alberta, Saskatchewan, Nova Scotia, Canada	[50]
Chaetoviridin F (**47**)	416	C_23_H_25_ClO_5_	*C. cochliodes* VTh01 *C. cochliodes* CTh05	Soil	Ubon Rajathanee province, Bangkok, Thailand Chiangrai province, Bangkok, Thailand	[60]
			*C. globosum*	*Viguiera robusta* (Leaves, Asteraceae)	Spain	[36]
4′-*Epi*-chaetoviridin F (**48**)	416	C_23_H_25_ClO_5_	*C. globosum*	*Viguiera robusta* (Leaves, Asteraceae)	Spain	[36]
Chaetoviridin G (**49**)	416	C_23_H_25_ClO_5_	*C. globosum*	*Viguiera robusta* (Leaves, Asteraceae)	Spain	[36]
Chaetoviridin G (**50**)	420	C_23_H_29_ClO_5_	*C. siamense*	Soil	Bangkok, Thailand	[62]
Chaetoviridin H (**51**)	398	C_23_H_26_ClO_6_	*C. globosum* CBS148.51	Cultured	China	[46]
			*C. globosum*	*Viguiera robusta* (Leaves, Asteraceae)	Spain	[36]
Chaetoviridin I (**52**)	466	C_23_H_27_ClO_8_	*C. globosum*	*Viguiera robusta* (Leaves, Asteraceae)	Spain	[36]
Chaetoviridin J (**53**)	408	C_22_H_29_ClO_5_	*C. globosum* TY1	*Ginkgo biloba* (Barks Ginkgoaceae)	Linyi, Shandong, China	[47]
			*C. globosum*	*Wikstroemia uva-ursi* (Leaves, Thymelaeaceae)	Hawaiian Islands, USA	[56]
			*C. globosum*	Seawater and marine deposits	Jeju, Korea	[54]
Chaetoviridin K (**54**)	450	C_23_H_27_ClO_7_	*C. globosum*	*Wikstroemia uva-ursi* (Leaves, Thymelaeaceae)	Hawaiian Islands, USA	[56]

**Table 2:** Biological activities of chaetomugilins and chaetoviridines.

Compound Name	Biological Activity	Assay, Organism, or Cell Line	Biological Results	Positive Control	Refs.
Chaetomugilin 106B-6 XXVIII (**1**)	Cytotoxicity	MTT/P388	32.0 µM (IC_50_)	5-FU 1.7 µM (IC_50_)	[37,58]
		MTT/HL-60	51.8 µM (IC_50_)	5-FU 2.7 µM (IC_50_)	[37,58]
		MTT/L1210	67.1 µM (IC_50_)	5-FU 1.1 µM (IC_50_)	[37,58]
		MTT/KB	58.8 µM (IC_50_)	5-FU 7.7 µM (IC_50_)	[37,58]
Chaetomugilin A (**2**)	Cytotoxicity	MTT/P388	8.7 µM (IC_50_)	5-FU 1.7 µM (IC_50_)	[38,40]
		MTT/HL-60	7.3 µM (IC_50_)	5-FU 2.7 µM (IC_50_)	[38,40]
		MTT/HL-60	6.4 µM (IC_50_)	Adriamycin 0.1 µM (IC_50_)	[22]
		MTT/K562	11.1 µM (IC_50_)	Adriamycin 0.3 µM (IC_50_)	[22]
		SRB/BEL-7402	17.9 µM (IC_50_)	Adriamycin 0.4 µM (IC_50_)	[22]
		SRB/HCT-116	6.1 µM (IC_50_)	Adriamycin 0.2 µM (IC_50_)	[22]
		SRB/HeLa	20.3 µM (IC_50_)	Adriamycin 0.6 µM (IC_50_)	[22]
		SRB/L-02	15.2 µM (IC_50_)	Adriamycin 0.4 µM (IC_50_)	[22]
		SRB/MGC-803	15.3 µM (IC_50_)	Adriamycin 0.2 µM (IC_50_)	[22]
		SRB/HO8910	12.1 µM (IC_50_)	Adriamycin 0.4 µM (IC_50_)	[22]
		SRB/SH-SY5Y	23.4 µM (IC_50_)	Adriamycin 0.2 µM (IC_50_)	[22]
		SRB/NCI-H1975	18.3 µM (IC_50_)	Adriamycin 0.3 µM (IC_50_)	[22]
		SRB/U87	27.1 µM (IC_50_)	Adriamycin 0.1 µM (IC_50_)	[22]
		SRB/MDA-MB-231	22.7 µM (IC_50_)	Adriamycin 0.2 µM (IC_50_)	[22]
11-*Epi*-chaetomugilin A (**4**)	Cytotoxicity	MTT/P388	88.9 µM (IC_50_)	5-FU 1.7 µM (IC_50_)	[35]
		MTT/HL-60	66.7 µM (IC_50_)	5-FU 2.7 µM (IC_50_)	[35]
		MTT/P388	88.9 µM (IC_50_)	5-FU 1.7 µM (IC_50_)	[37,58]
		MTT/HL-60	66.7 µM (IC_50_)	5-FU 2.7 µM (IC_50_)	[37,58]
		MTT/L1210	80.2 µM (IC_50_)	5-FU 1.1 µM (IC_50_)	[37,58]
Chaetomugilin B (**6**)	Cytotoxicity	MTT/P388	18.7 µM (IC_50_)	5-FU 1.7 µM (IC_50_)	[38,40]
		MTT/HL-60	16.5 µM (IC_50_)	5-FU 2.7 µM (IC_50_)	[38,40]
Chaetomugilin C (**7**)	Cytotoxicity	MTT/P388	3.6 µM (IC_50_)	5-FU 1.7 µM (IC_50_)	[38,40]
		MTT/HL-60	2.7 µM (IC_50_)	5-FU 2.7 µM (IC_50_)	[38,40]
		MTT/HL-60	6.6 µM (IC_50_)	Adriamycin 0.1 µM (IC_50_)	[22]
		MTT/K562	12.3 µM (IC_50_)	Adriamycin 0.3 µM (IC_50_)	[22]
		SRB/BEL-7402	16.8 µM (IC_50_)	Adriamycin 0.4 µM (IC_50_)	[22]
		SRB/HCT-116	5.7 µM (IC_50_)	Adriamycin 0.2 µM (IC_50_)	[22]
		SRB/HeLa	13.2 µM (IC_50_)	Adriamycin 0.6 µM (IC_50_)	[22]
		SRB/L-02	9.1 µM (IC_50_)	Adriamycin 0.4 µM (IC_50_)	[22]
		SRB/MGC-803	9.6 µM (IC_50_)	Adriamycin 0.2 µM (IC_50_)	[22]
		SRB/HO8910	8.8 µM (IC_50_)	Adriamycin 0.4 µM (IC_50_)	[22]
		SRB/SH-SY5Y	19.4 µM (IC_50_)	Adriamycin 0.2 µM (IC_50_)	[22]
		SRB/NCI-H1975	12.1 µM (IC_50_)	Adriamycin 0.3 µM (IC_50_)	[22]
		SRB/U87	17.6 µM (IC_50_)	Adriamycin 0.1 µM (IC_50_)	[22]
		SRB/MDA-MB-231	26.6 µM (IC_50_)	Adriamycin 0.2 µM (IC_50_)	[22]
Chaetomugilin D (**8**)	Cytotoxicity	MTT/P388	7.5 µM (IC_50_)	5-FU 1.7 µM (IC_50_)	[38,40]
		MTT/HL-60	6.8 µM (IC_50_)	5-FU 2.7 µM (IC_50_)	[38,40]
	Phytotoxic activity	Lettuce seed germination bioassay/Root growth inhibition	24.2 ppm (IC_50_)	-	[51]
		Lettuce seed germination bioassay/Shoot growth inhibition	27.8 ppm (IC_50_)	-	[51]
	Antimicrobial	Microplate assay/*Vibrio vulnificus*	32.4 µg/mL (MIC)	Erythromycin 2.0 µg/mL (MIC)	[25]
		Microplate assay/*Vibrio rotiferianus*	15.3 µg/mL (MIC)	Erythromycin 3.9 µg/mL (MIC)	[25]
		Microplate assay/MRSA 1	32.2 µg/mL (MIC)	Chloramphenicol 7.6 µg/mL (MIC)	[25]
		Microplate assay/MRSA 2	32.4 µg/mL (MIC)	Chloramphenicol 7.5 µg/mL (MIC)	[25]
*Seco*-chaetomugilin D (**9**)	Cytotoxicity	MTT/P388	38.6 µM (IC_50_)	5-FU 1.7 µM (IC_50_)	[48]
		MTT/HL-60	47.2 µM (IC_50_)	5-FU 2.7 µM (IC_50_)	[48]
		MTT/L1210	53.6 µM (IC_50_)	5-FU 3.0 µM (IC_50_)	[48]
		MTT/KB	47.2 µM (IC_50_)	5-FU 6.0 µM (IC_50_)	[48]
Chaetomugilin E (**11**)	Cytotoxicity	MTT/P388	15.7 µM (IC_50_)	5-FU 1.7 µM (IC_50_)	[38,40]
		MTT/HL-60	13.2 µM (IC_50_)	5-FU 2.7 µM (IC_50_)	[38,40]
	Inhibition TNF-α	TNF-α activated NF-kB assay	11.6 µM (IC_50_)	TPCK 3.8 µM (IC_50_) BAY-11 2.0 µM (IC_50_)	[56]
	Inhibition NO	Nitrite assay	5.8 µM (IC_50_)	L-NMMA 25.1 µM (IC_50_)	[56]
Chaetomugilin F (**13**)	Cytotoxicity	MTT/P388	3.3 µM (IC_50_)	5-FU 1.7 µM (IC_50_)	[38,40]
		MTT/HL-60	1.3 µM (IC_50_)	5-FU 2.7 µM (IC_50_)	[38,40]
	Inhibition TNF-α	TNF-α activated NF-kB assay	5.1 µM (IC_50_)	TPCK 3.8 µM (IC_50_) BAY-11, 2.0 µM (IC_50_)	[56]
	Inhibition NO	Nitrite assay	1.9 µM (IC_50_)	L-NMMA 25.1 µM (IC_50_)	[56]
Chaetomugilin G (**14**)	Cytotoxicity	MTT/P388	24.1 µM (IC_50_)	5-FU 1.7 µM (IC_50_)	[38]
		MTT/HL-60	19.8 µM (IC_50_)	5-FU 2.7 µM (IC_50_)	[38]
		MTT/P388	24.1 µM (IC_50_)	5-FU 1.7 µM (IC_50_)	[41]
		MTT/HL-60	19.8 µM (IC_50_)	5-FU 2.7 µM (IC_50_)	[41]
		MTT/L1210	123.6 µM (IC_50_)	5-FU 3.0 µM (IC_50_)	[41]
		MTT/KB	137.8 µM (IC_50_)	5-FU 6.0 µM (IC_50_)	[41]
Chaetomugilin H (**15**)	Cytotoxicity	MTT/P388	12.3 µM (IC_50_)	5-FU 1.7 µM (IC_50_)	[38]
		MTT/HL-60	10.3 µM (IC_50_)	5-FU 2.7 µM (IC_50_)	[38]
		MTT/P388	12.3 µM (IC_50_)	5-FU 1.7 µM (IC_50_)	[41]
		MTT/HL-60	10.3 µM (IC_50_)	5-FU 2.7 µM (IC_50_)	[41]
		MTT/L1210	93.3 µM (IC_50_)	5-FU 3.0 µM (IC_50_)	[41]
		MTT/KB	18.8 µM (IC_50_)	5-FU 6.0 µM (IC_50_)	[41]
Chaetomugilin I (16)	Cytotoxicity	MTT/P388	1.1 µM (IC_50_)	5-FU 1.7 µM (IC_50_)	[37,58,59]
		MTT/HL-60	1.1 µM (IC_50_)	5-FU 2.7 µM (IC_50_)	[37,58,59]
		MTT/L1210	1.9 µM (IC_50_)	5-FU 1.1 µM (IC_50_)	[37,58,59]
		MTT/KB	2.3 µM (IC_50_)	5-FU 7.7 µM (IC_50_)	[37,58,59]
	Inhibition TNF-α	TNF-α activated NF-kB assay	0.9 µM (IC_50_)	TPCK 3.8 µM (IC_50_) BAY-11 2.0 µM (IC_50_)	[56]
	Inhibition NO	Nitrite assay	0.3 µM (IC_50_)	L-NMMA 25.1 µM (IC_50_)	[56]
11-*Epi*-chaetomugilin I (**17**)	Cytotoxicity	MTT/P388	0.7 µM (IC_50_)	5-FU 1.7 µM (IC_50_)	[59]
		MTT/HL-60	1.0 µM (IC_50_)	5-FU 2.7 µM (IC_50_)	[59]
		MTT/L1210	1.6 µM (IC_50_)	5-FU 1.1 µM (IC_50_)	[59]
		MTT/KB	1.2 µM (IC_50_)	5-FU 7.7 µM (IC_50_)	[59]
	Inhibition TNF-α	TNF-α activated NF-kB assay	0.9 µM (IC_50_)	TPCK 3.8 µM (IC_50_) BAY-11 2.0 µM (IC_50_)	[56]
	Inhibition NO	Nitrite assay	0.8 µM (IC_50_)	L-NMMA 25.1 µM (IC_50_)	[56]
Chaetomugilin J (**18**)	Phytotoxic activity	Lettuce seed germination bioassay/Root growth inhibition	22.6 ppm (IC_50_)	-	[51]
		Lettuce seed germination bioassay/Shoot growth inhibition	21.9 ppm (IC_50_)	-	[51]
	Cytotoxicity	MTT/P388	12.6 µM (IC_50_)	5-FU 1.7 µM (IC_50_)	[37,58]
		MTT/HL-60	12.6 µM (IC_50_)	5-FU 2.7 µM (IC_50_)	[37,58]
		MTT/L1210	2.8 µM (IC_50_)	5-FU 1.1 µM (IC_50_)	[37,58]
		MTT/KB	8.5 µM (IC_50_)	5-FU 7.7 µM (IC_50_)	[37,58]
	Inhibition TNF-α	TNF-α activated NF-kB assay	7.6 µM (IC_50_)	TPCK 3.8 µM (IC_50_) BAY-11 2.0 µM (IC_50_)	[56]
	Inhibition NO	Nitrite assay	4.2 µM (IC_50_)	L-NMMA 25.1 µM (IC_50_)	[56]
Chaetomugilin K (**19**)	Cytotoxicity	MTT/P388	8.2 µM (IC_50_)	5-FU 1.7 µM (IC_50_)	[58]
		MTT/HL-60	14.1 µM (IC_50_)	5-FU 2.7 µM (IC_50_)	[58]
		MTT/L1210	11.2 µM (IC_50_)	5-FU 1.1 µM (IC_50_)	[58]
		MTT/KB	18.7 µM (IC_50_)	5-FU 7.7 µM (IC_50_)	[58]
Chaetomugilin L (**20**)	Cytotoxicity	MTT/P388	10.9 µM (IC_50_)	5-FU 1.7 µM (IC_50_)	[58]
		MTT/HL-60	13.1 µM (IC_50_)	5-FU 2.7 µM (IC_50_)	[58]
		MTT/L1210	15.6 µM (IC_50_)	5-FU 1.1 µM (IC_50_)	[58]
		MTT/KB	20.1 µM (IC_50_)	5-FU 7.7 µM (IC_50_)	[58]
Chaetomugilin N (**22**)	Cytotoxicity	MTT/P388	2.3 µM (IC_50_)	5-FU 1.7 µM (IC_50_)	[58]
		MTT/HL-60	2.3 µM (IC_50_)	5-FU 2.7 µM (IC_50_)	[58]
		MTT/L1210	10.6 µM (IC_50_)	5-FU 1.1 µM (IC_50_)	[58]
		MTT/KB	10.6 µM (IC_50_)	5-FU 7.7 µM (IC_50_)	[58]
Chaetomugilin O (**23**)	Cytotoxicity	MTT/P388	11.1 µM (IC_50_)	5-FU 1.7 µM (IC_50_)	[58]
		MTT/HL-60	11.1 µM (IC_50_)	5-FU 2.7 µM (IC_50_)	[58]
		MTT/L1210	10.1 µM (IC_50_)	5-FU 1.1 µM (IC_50_)	[58]
		MTT/KB	7.2 µM (IC_50_)	5-FU 7.7 µM (IC_50_)	[58]
Chaetomugilin P (**24**)	Cytotoxicity	MTT/P388	0.7 µM (IC_50_)	5-FU 1.7 µM (IC_50_)	[59]
		MTT/HL-60	1.2 µM (IC_50_)	5-FU 2.7 µM (IC_50_)	[59]
		MTT/L1210	1.5 µM (IC_50_)	5-FU 1.1 µM (IC_50_)	[59]
		MTT/KB	1.8 µM (IC_50_)	5-FU 7.7 µM (IC_50_)	[59]
Chaetomugilin Q (**25**)	Cytotoxicity	MTT/P388	49.5 µM (IC_50_)	5-FU 1.7 µM (IC_50_)	[59]
		MTT/HL-60	47.2 µM (IC_50_)	5-FU 2.7 µM (IC_50_)	[59]
		MTT/L1210	80.2 µM (IC_50_)	5-FU 1.1 µM (IC_50_)	[59]
Chaetomugilin R (**26**)	Cytotoxicity	MTT/P388	32.0 µM (IC_50_)	5-FU 1.7 µM (IC_50_)	[59]
		MTT/HL-60	51.8 µM (IC_50_)	5-FU 2.7 µM (IC_50_)	[59]
		MTT/L1210	67.1 µM (IC_50_)	5-FU 1.1 µM (IC_50_)	[59]
		MTT/KB	67.1 µM (IC_50_)	5-FU 7.7 µM (IC_50_)	[59]
Chaetomugilin S (**27**)	Caspase-3 inhibitory	Caspase-3 enzymatic assay	20.6 µM (IC_50_)	Ac-DEVD-CHO 13.7 nM (IC_50_)	[31]
Chaetomugilin T (**29**)	Cytotoxicity	MTT/P388	62.4 µM (IC_50_)	5-FU 1.9 µM (IC_50_)	[43]
		MTT/HL-60	67.2 µM (IC_50_)	5-FU 2.3 µM (IC_50_)	[43]
Chaetomugilin U (**30**)	Cytotoxicity	MTT/P388	57.4 µM (IC_50_)	5-FU 1.9 µM (IC_50_)	[43]
		MTT/HL-60	57.4 µM (IC_50_)	5-FU 2.3 µM (IC_50_)	[43]
		MTT/L1210	94.8 µM (IC_50_)	5-FU 2.2 µM (IC_50_)	[43]
Chaetoviridin A (**31**)	Antifungal	*Alternaria mali*	33.3 µg/mL (MIC)	-	[61]
		*Botrytis cinerea*	33.3 µg/mL (MIC)	-	[61]
		*Colletotrichum gloeosporioides*	33.3 µg/mL (MIC)	-	[61]
		*Fusarium oxysporum*	33.3 µg/mL (MIC)	-	[61]
		*Phytophthora capsici*	33.3 µg/mL (MIC)	-	[61]
		*Phytophthora infestans*	33.3 µg/mL (MIC)	-	[61]
		*Pythium ultimum*	1.23 µg/mL (MIC)	-	[61]
		*Magnaporthe grisea*	1.23 µg/mL (MIC)	-	[61]
		*Sclerotinia sclerotiorum*	97.5 (% inhibition)	-	[73]
		*Botrytis cinerea*	69.1 (% inhibition)	-	[73]
		*Fusarium graminearum*	77.0 (% inhibition)	-	[73]
		*Phytophthora capsici*	60.7 (% inhibition)	-	[73]
		*Fusarium moniliforme*	59.2 (% inhibition)	-	[73]
5′-*Epi*-chaetoviridin A (**33**)	Cytotoxicity	SRB assay/HepG-2	35.3 µM (IC_50_)	Camptothecin 32.3 µM (IC_50_)	[72]
7,5′- *Bis*-*epi*-chaetoviridin A (**34**)	Caspase-3 inhibitory	Caspase-3 enzymatic assay	10.9 µM (IC_50_)	Ac-DEVD-CHO 13.7 nM (IC_50_)	[31]
*N*-glutarylchaetoviridin A (**35**)	Cytotoxicity	MTT/HL-60	10.3 µM (IC_50_)	Adriamycin 0.1 µM (IC_50_)	[22]
		MTT/K562	20.3 µM (IC_50_)	Adriamycin 0.3 µM (IC_50_)	[22]
		SRB/BEL-7402	23.9 µM (IC_50_)	Adriamycin 0.4 µM (IC_50_)	[22]
Chaetoviridin B (**37**)	Antifungal	*Pythium ultimum*	33.3 µg/mL (MIC)	-	[61]
		*Magnaporthe grisea*	33.3 µg/mL (MIC)	-	[61]
Chaetoviridin B (**38**)	α-Glucosidase inhibiory	Spectrophotometric assay	6.328 µM (IC_50_)	Acarbose 54.74 µM (IC_50_)	[47]
*N*-Glutarylchaetoviridin C (**42**)	Cytotoxicity	MTT/HL-60	11.1 µM (IC_50_)	Adriamycin 0.1 µM (IC_50_)	[22]
		MTT/K562	11.7 µM (IC_50_)	Adriamycin 0.3 µM (IC_50_)	[22]
		SRB/BEL-7402	10.9 µM (IC_50_)	Adriamycin 0.4 µM (IC_50_)	[22]
		SRB/HCT-116	11.3 µM (IC_50_)	Adriamycin 0.2 µM (IC_50_)	[22]
		SRB/HeLa	22.1 µM (IC_50_)	Adriamycin 0.6 µM (IC_50_)	[22]
		SRB/L-02	18.2 µM (IC_50_)	Adriamycin 0.4 µM (IC_50_)	[22]
		SRB/MGC-803	6.6 µM (IC_50_)	Adriamycin 0.2 µM (IC_50_)	[22]
		SRB/HO8910	9.7 µM (IC_50_)	Adriamycin 0.4 µM (IC_50_)	[22]
		SRB/NCI-H1975	11.2 µM (IC_50_)	Adriamycin 0.3 µM (IC_50_)	[22]
		SRB/U87	18.3 µM (IC_50_)	Adriamycin 0.1 µM (IC_50_)	[22]
		SRB/MDA-MB-231	13.2 µM (IC_50_)	Adriamycin 0.2 µM (IC_50_)	[22]
Chaetoviridin E (**44**)	Cytotoxicity	SRB assay/BC1	5.6 µg/mL (IC_50_)	Ellipticine 0.26 µg/mL (IC_50_)	[60]
	Cytotoxicity	SRB assay/NCl-H187	3.5 µg/mL (IC_50_)	Ellipticine 0.32 µg/mL (IC_50_)	[60]
	Cytotoxicity	SRB assay/HepG-2	40.6 µM (IC_50_)	Camptothecin 32.3 µM (IC_50_)	[72]
	Antimalarial	Microculture radioisotope assay/*P. falciparum* (K1, MDR)	2.9 µg/mL (IC_50_)	-	[60]
7-*Epi*-chaetoviridin E (**45**)	Caspase-3 inhibitory	Caspase-3 enzymatic assay	7.9 µM (IC_50_)	Ac-DEVD-CHO 13.7 nM (IC_50_)	[31]
Chaetoviridin F (**47**)	Cytotoxicity	SRB assay/NCl-H187	4.5 µg/mL (IC_50_)	Ellipticine 0.32 µg/mL (IC_50_)	[60]
	Cytotoxicity	MTT/P388	46.0 µM (IC_50_)	5-FU 1.9 µM (IC_50_)	[43]
		MTT/HL-60	39.1 µM (IC_50_)	5-FU 2.3 µM (IC_50_)	[43]
		MTT/L1210	43.7 µM (IC_50_)	5-FU 2.2 µM (IC_50_)	[43]
		MTT/KB	34.5 µM (IC_50_)	5-FU 20.6 µM (IC_50_)	[43]

The plates were developed by using toluene/EtOAc/formic acid (7:3:1), CH_2_Cl_2_/MeOH (20:1), benzene/ethyl acetate (8:2), EtOAc/CH_2_Cl_2_ (5:95), n-hexane/ethyl acetate (4:1), or EtOAc/CH_2_Cl_2_ (2:8) [29,42,60,63]. The isolated metabolites can be purified by recrystallization from MeOH or CHCl_3_:MeOH until they show constant melting points.

The structures of isolated metabolites were determined through extensive spectroscopic analyses, including UV, IR, MS, and 1D (^1^H, ^13^C NMR, and DEPT) and 2D NMR (COSY, NOESY, ROESY, HMQC, HSQC, or HMBC).

The absolute configurations of these metabolites have been established with the aid of optical rotation sign, X-ray crystallography, CD (circular dichroism), the modified Mosher’s method, and chemical transformation studies, including derivatization and degradation [22,27,35,41,48,60,75]. It has been reported that the absolute configuration at C(7) controls signs of the specific rotation [35]. Compounds with (S) C-11 and C-7 had negative optical rotation values; however, when C-7 was (R), the sign switched to positive with the same magnitude [50]. The absolute (S) configuration at C-7 was determined by the negative Cotton effect in the CD spectrum [58]. Mass spectra of these compounds displayed an isotopic peak [M+H]^+^/[M+H+2]^+^ in a ratio 3:1, characterizing the presence of a single chlorine atom. Moreover, their IR spectrum exhibited characteristic bands for a hydroxyl group (3405–3450 cm^−1^), lactone (1718–1780 cm^−1^), and α,β-unsaturated ketone (1616–1684 cm^−1^). Characteristic UV bands of a highly extended conjugation system were observed at 283–429 nm.

## 3. Biological Activities

### 3.1. Cytotoxic Activity

**Figure 2:**
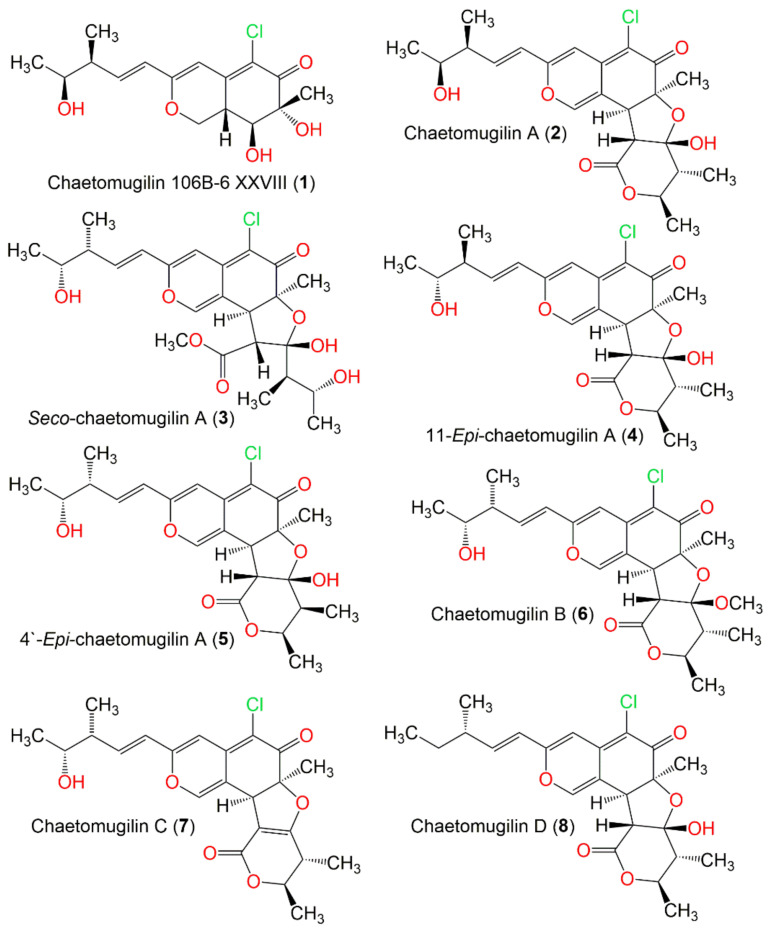
Structures of compounds **1**–**8**.

**Figure 3:**
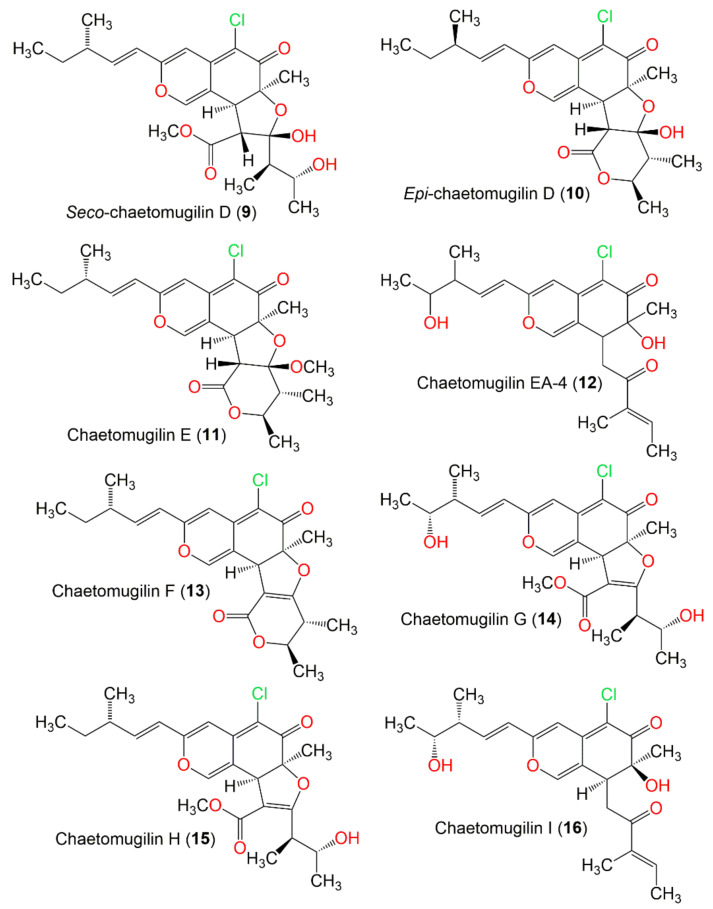
Structures of compounds **9**–**16**.

Yamada et al. reported the isolation of chaetomugilins A (**2**), B (**6**), C (**7**), D (**8**), E (**11**), F (**13**), G (**14**), and H (**15**) from the culture broth of *C. globosum* associated with marine fish *Mugil cephalus* and assessed for their cytotoxic effects on P388 and HL-60 cell lines in the MTT assay (Figure 2 and Figure 3; Table 2). It is noteworthy that compounds **7** and **13** exhibited remarkable cytotoxicity towards P388 and HL-60 cell lines (IC_50_ 3.6 and 3.3 µM and 2.7 and 1.3 µM, respectively), nearly equal to that of 5-fluorouracil (IC_50_ 1.7 and 2.7 µM, respectively). While other compounds had moderate to weak cytotoxicity (IC_50_ ranging from 6.8 to 24.1 µM) [38,39]. Further, **2**, **7**, and **13** displayed selective cytotoxicity towards a panel of 39 disease-related human cell lines, including breast, CNS, colon, lung, melanoma, ovary, kidney, stomach, and prostate cancer cells with range and delta values of **2** (1.24 and 1.13, respectively), **7** (1.19 and 0.71, respectively), and **13** (1.21 and 1.97, respectively) [38,40]. It was suggested that the existence of C-12-hydroxyl and C-3-methoxyl groups had little effect on the activity [40]. Evaluation of the differential cytotoxicity patterns using COMPARE revealed that the modes of action for **2**, **7**, and **13** might be different from those of other anticancer drugs [40]. On the other hand, chaetomugilins A (**2**) biosynthesized by *C. globosum* Z1 isolated from *Broussonetia papyrifera* bark had no in vitro effectiveness towards SMMC-7721, MG-63 and A-549 cell lines (IC_50_ ˃ 50 µg/mL) in the MTT assay in comparison to doxorubicin [44].

*C*. *globosum* OUPS-T106B-6 isolated from *M. cephalus* yielded two new metabolites that demonstrated moderate cytotoxicity towards HL-60 and P388 cell lines (IC_50_ ranged from 10.3 to 24.1 µM, respectively), compared to 5-FU (IC_50_ 2.7 and 1.7 µM) in the MTT assay [41]: chaetomugilins G (**14**) and H (**15**).

In another study by Yamada et al. on the same fungus, two new compounds named *seco*-chaetomugilins A (**3**) and D (**8**) were separated. Compound **8** exhibited weak activity (IC50 38.6–53.6 μM) towards HL-60, P388, KB, and L1210, compared with 5-FU (IC_50_ 1.7–6.0 μM); however, **8** was inactive. It was suggested that the C-12-hydroxyl group decreased the activity [48].

Chaetomugilins I (**16**), J (**18**), 11-epichaetomugilin A (**4**), 4′-epichaetomugilin A (**5**), and 106B-6 XXVIII (**1**) were separated from *C. globosum* 106B-6 and assessed for cytotoxic activity towards P388, HL-60, L1210, and KB cell lines. Interestingly, compounds **16** and **18** had significant cytotoxicity (IC_50_ 1.1–2.3 µM for **16** and 2.8–12.8 µM for **18**) towards all cell lines equal to that of 5-FU (IC_50_ 1.1–7.7 µM). In addition, **16** showed potent and selective cytotoxic activity towards a panel of 39 human cell lines. The other compounds exhibited moderate to marginal activity toward the tested cell lines [37].

Muroga et al. assessed the cytotoxicity of the new metabolites, chaetomugilins I–O (**16** and **18**–**23**), towards P388, HL-60, L1210, and KB cell lines using the MTT assay. Compounds **16**, **18**–**20**, **22**, and **23** revealed remarkable cytotoxicity (IC_50_ ranged from 1.1 to 20.1 µM) towards all cell lines, compared to 5-FU (IC_50_ 1.1–7.7 µM), whereas chaetomugilin M (**21**) was inactive. Particularly, compound **16** was more potent than 5-FU. Further, **16** had selective and potent cytotoxicity towards a panel of 39 human cell lines [58].

Furthermore, the new metabolites, 11- and 4′-epichaetomugilin A (**4** and **5**) purified from *C. globosum* isolated from *M. cephalus*, displayed moderate to weak cytotoxicity toward KB, P388, HL-60, and L1210 cell lines [35].

**Figure 4:**
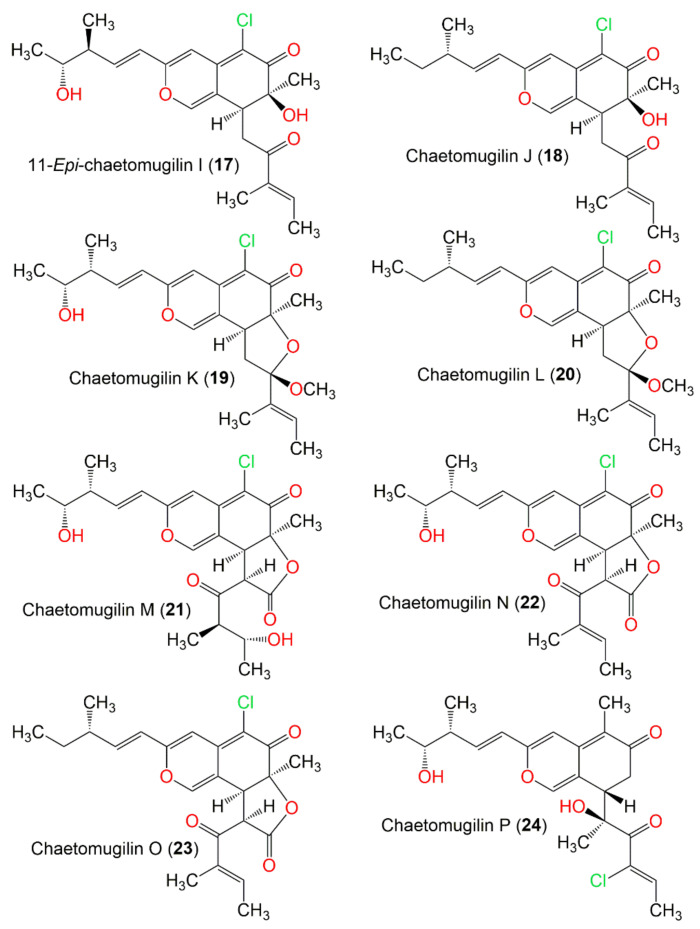
Structures of compounds **17**–**24**.

**Figure 5:**
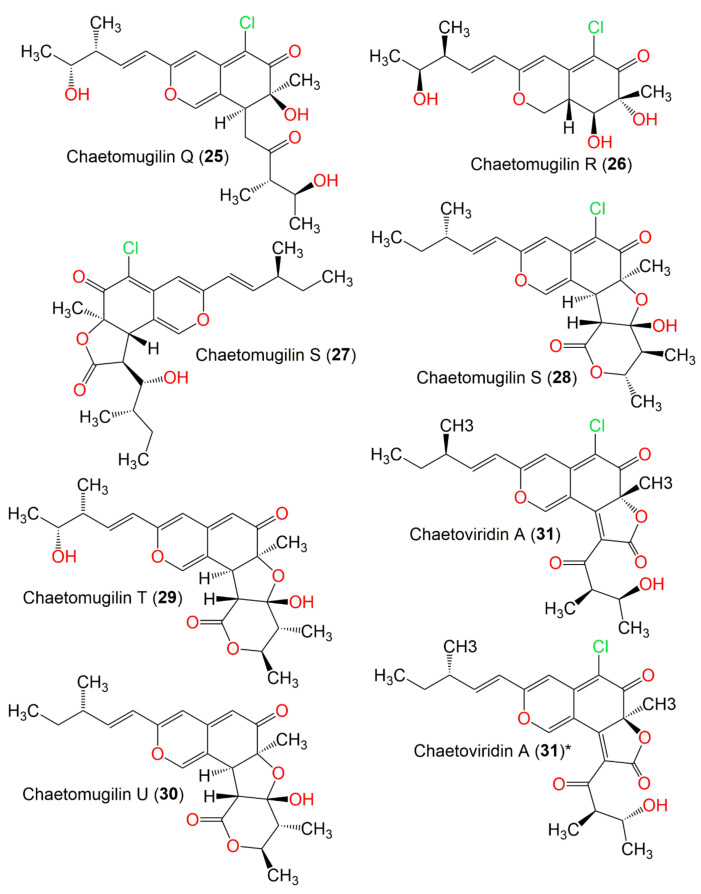
Structures of compounds **25**–**31** (* Revised by Makrerougras et al).

The new metabolites, chaetomugilins P–R (**24**–**27**) and 11-*epi*-chaetomugilin I (**17**), along with the formerly separated chaetomugilin I (**16**), were purified by the marine fish-associated *C. globosum* (Figure 4 and Figure 5) [59]. Compounds **24**, **17**, and **16** possessed stronger cytotoxicity towards HL-60, P388, KB, and L1210 (IC_50_ 1.1, 1.1, 2.3, and 1.9 μM, respectively, for **16**; 1.0, 0.7, 1.2, and 1.6 μM, respectively, for **17**; and 1.2, 0.7, 1.8, and 1.5 μM, respectively, for **24**) cell lines than 5-FU (IC_50_ 2.7, 1.7, 7.7, and 1.1 μM, respectively). The results indicated that the C-2′–C-4′ enone moiety is essential for activity. On the other hand, compounds **25**–**27** were weakly to moderately active towards all tested cancer cell lines (IC_50_ 32.0–80.2 μM) [59].

The new metabolites, chaetomugilin S (**28**), T (**29**), and U (**30**), separated from *C*. *globosum* derived from *M. cephalus*, revealed moderate to high growth inhibition towards HL-60, P388, KB, and L1210 cell lines (IC_50_ ranging from 34.4 to 94.9 µM), relative to 5-FU (IC_50_ 1.9–20.6 µM) [43].

**Figure 6:**
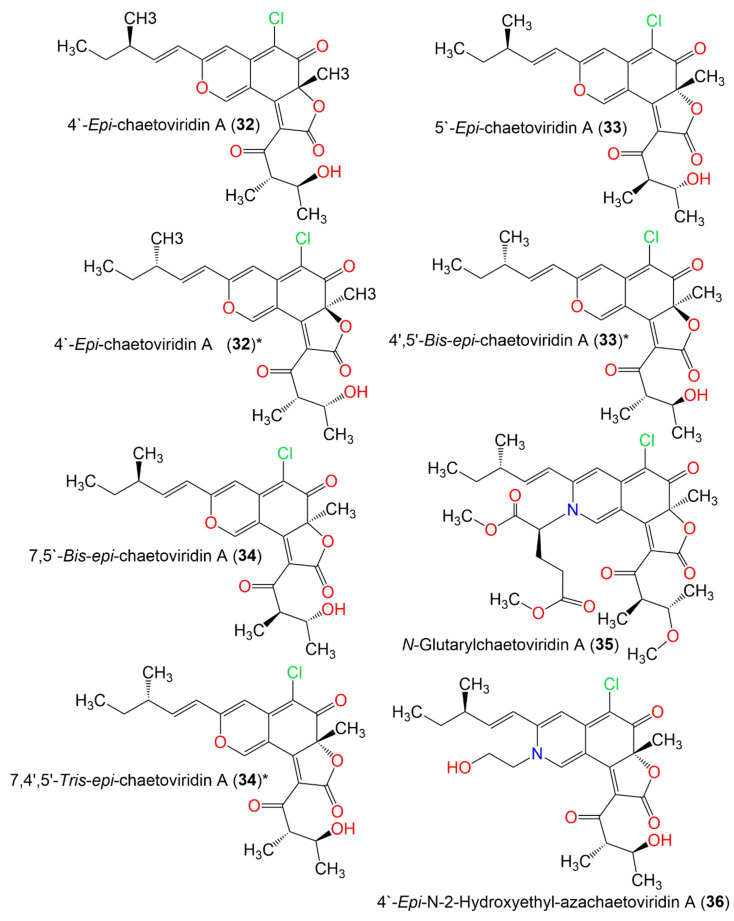
Structures of compounds **32**–**36** (* Revised and renamed by Makrerougras et al.).

In 2019, Sun et al. evaluated the cytotoxicity of the new glutamine-containing derivatives, *N*-glutarylchaetoviridins A–C (**35**, **39**, and **42**), in addition to chaetomugilins A (**2**) and C (**7**) from the extract of deep-sea sediment-associated *C. globosum* HDN151398 toward BEL-7402, HeLa, HCT-116, L-02, HO8910, MGC-803, SH-SY5Y, U87, NCI-H1975, and MDA-MB-231 cancer cells using the SRB method and towards HL-60 and K562 using MTT method (Figure 6). Chaetomugilins A (**2**) and C (**7**) and N-glutarylchaetoviridin C (**42**) exhibited powerful cytotoxicity towards all tested cell lines (IC_50_ 5.7–27.1 µM for **2**, 6.6–26.6 µM for **7**, and 6.6–26.5 µM for **42)** compared to adriamycin (IC_50_ 0.1–0.6 µM) [22]. Among them, N-glutarylchaetoviridin C (**42**) had a remarkable cytotoxicity toward MGC-803 and HO-8910 (IC_50_ 6.6 and 9.7 µM, respectively) [22].

Chaetomugilin A (**2**), 11-*epi*-chaetomugilin A (**4**), and chaetomugilin D (**8**) displayed no noticeable cytotoxic activity (IC_50_ ˃ 40 μM) toward HepG-2, A549, and HeLa in the MTT assay compared to etoposide (IC_50_ 16.11, 16.46, and 15.00 μM, respectively) [46].

Wani et al. reported that *seco*-chaetomugilin D (**9**) isolated from *C. cupreum* had cytotoxicity towards MCF-7 (inhibition ranging from 25.25% to 75.25% after 24 h and from 41.5% to 99% after 38 h at 3.12–50 µg/mL). Further, it increased mitochondrial membrane depolarization (16.45% and 32.25% at 5 and 15 µg/mL, respectively) and induced ROS production (19.6% and 26.2% at 5 and 15 µg/mL, respectively) in comparison to untreated cells. Therefore, it caused cell death via induction of mitochondrial ROS production and membrane depolarization [29].

*C. globosum* isolated from *Ginkgo biloba* leaves yielded chaetomugilin D (**8**) and chaetomugilin A (**2**), which demonstrated significant toxicity toward brine shrimp (*Artemia salina*) larvae after 24 h (mortality rates 75.2 and 78.3%, respectively, at 10 µg/mL) [42].

**Figure 7:**
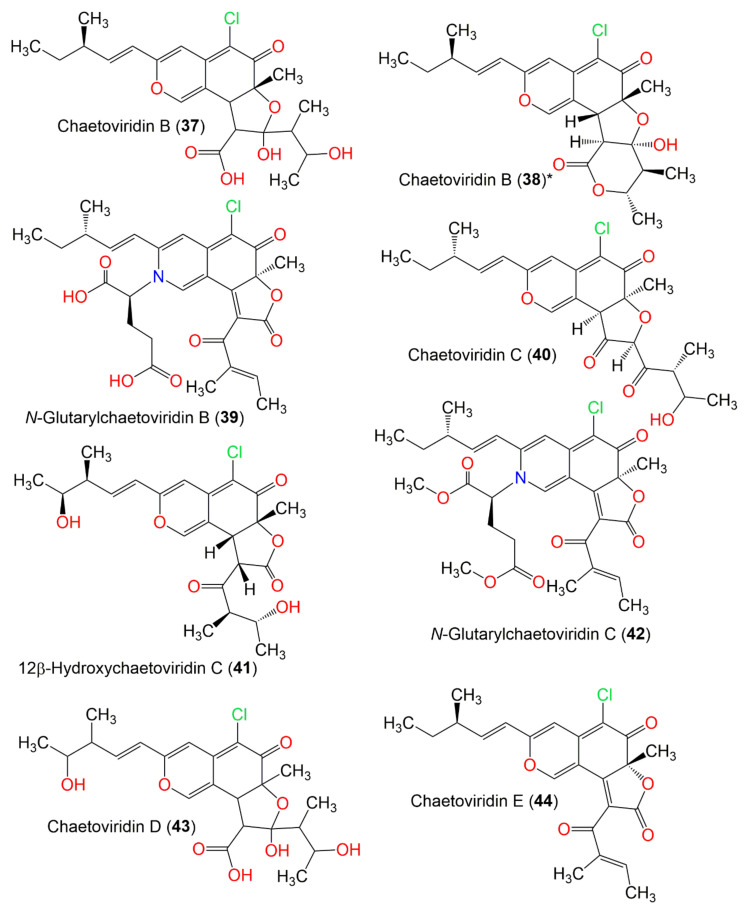
Structures of compounds **37**–**44** (* Revised Kingsland and Barrow).

Hu et al. proved that chaetomugilin J (**18**) combined with low-dose cisplatin decreased cell viability and boosted cisplatin-produced apoptosis in ovarian A2780 cells independently of the endoplasmic reticulum apoptotic pathway. It significantly induced mitochondrial dysfunction and apoptosis via increasing the intracellular and mitochondrial ROS levels and decreasing mitochondrial membrane potential. It also prohibited parkin/PINK1 induced mitophagy, resulting in weakening the mitophagy protective effect that led to apoptosis and increased sensitivity to cisplatin [34]. Chaetomugilin D (**8**), chaetoviridin A (**31**), and chaetoviridin E (**44**) purified from a deep sea-derived *Chaetomium* sp. NA-S01-R1 displayed moderate to weak cytotoxicity toward HeLa, A549, and HepG2, in comparison to doxorubicin (IC_50_ 0.1–1.1 µM) using the CCK-8 assay [25] (Figure 7). Additionally, chaetoviridin A (**31**) and chaetoviridin E (**44**) were inactive towards AGS and MGC803 [71].

In addition, chaetoviridins E and F (**44** and **47**) had cytotoxicity toward NCI-H187, KB, and BC1 (IC_50_ ranging from 3.5 to 13.4 µg/mL), in comparison to ellipticine (IC_50_ ranging from 0.26 to 0.36 µg/mL) [60].

Chaetoviridin A (**31**) (2 µM, ID_50_ 0.6 μM) inhibited the inflammatory activity of TPA (12-O-tetradecanoylphorbol-13-acetate, 1 µg) in mice (ID_50_ 0.6 μM). Furthermore, it markedly suppressed the promoting effect of TPA on skin tumor formation in mice initiated with 7,12-dimethylbenz[a]an-thracene (50 μg). It was proposed that it inhibited TPA-tumor promotion in a two-stage carcinogenesis model in mice due to its anti-inflammatory potential [76].

*C. globosum* TY1 associated with *Ginkgo biloba* barks yielded chaetoviridins B (**38**) and E (**44**) and 5′-epi-chaetoviridin A (**33**). Compounds **44** and **33** had moderate cytotoxicity towards HepG-2 (IC_50_ 40.6 and 35.3 µM, respectively) compared to camptothecin (IC_50_ 32.3 µM) in the SRB assay, and **38** was inactive [72].

*C. globosum* isolated from *Artemisia desterorum* roots yielded chaetoviridin E (**44**) and chaetoviridin A (**31**). They had no cytotoxic activity toward A549, HCT116, and HepG2 cancer cells [68]. The new metabolite, epi-chetomugilin D (**10**), along with chaetovirdins A (**31**), B (**37**), and E (**44**) and chetomugilins D (**8**) and J (**18**) were purified from *C. globosum* associated with *Adiantum capillus-veneris*. Chaetovirdin E (5 µg/mL) exhibited cytotoxicity towards CaCO_2_ and HepG2 cancers cells with 30% and 59% inhibition, respectively [49].

### 3.2. Antimicrobial Activity

**Figure 8:**
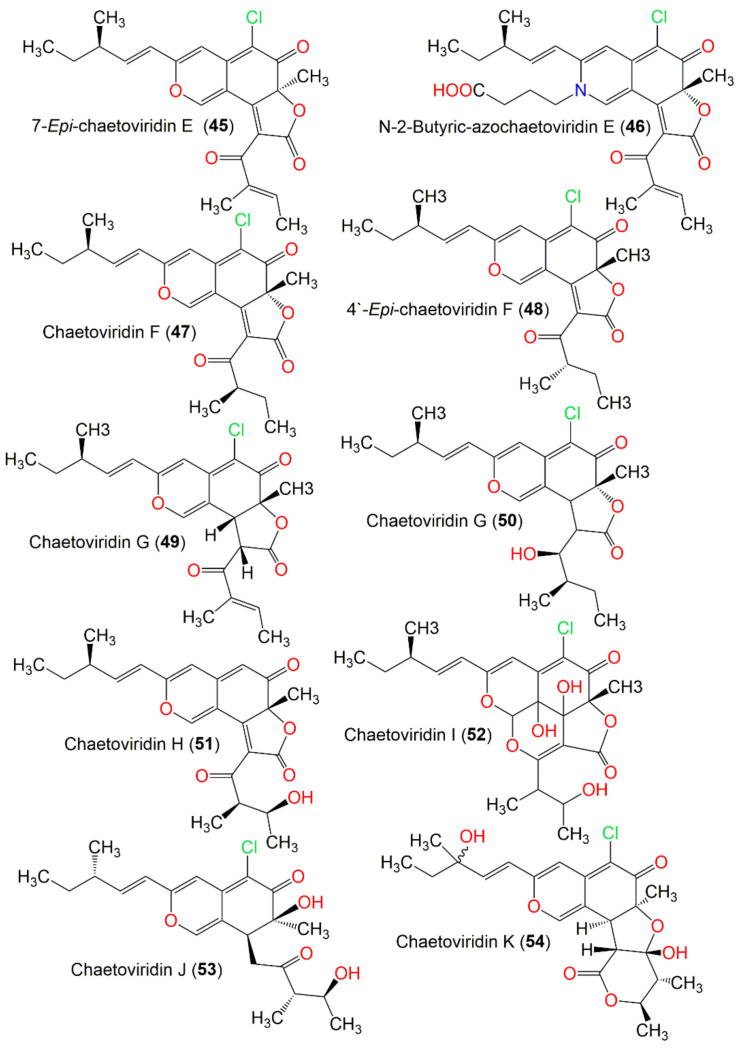
Structures of compounds **45**–**54**.

The liquid culture of *C. globosum* DAOM-240359 isolated from an indoor air sample collected from Ottawa, Ontario, Canada produced new nitrogen-containing chaetoviridins, 4′-epi-N-2-hydroxyethyl-azachaetoviridin A (**36**) and N-2-butyric-azochaetoviridin E (**46**), along with chaetoviridin A (**31**) and chaetomugilin D (**8**). Compound **36** is a nitrogenous derivative of **31** with an N-2-hydroxy ethyl chain and (R) configuration at C-4′ instead of the (S) configuration of **31**. Compound **46** is a nitrogenous derivative of chaetoviridin E (**44**) with a C-2 γ-aminobutyric acid moiety. This represents the first reported azaphilone with a 3-methyl-1-pentyl group and an N-2 side chain. Compounds **8** and 46 significantly reduced the growth of *Pseudomonas putida* and *B. subtilus* (conc. 20 µM) in the quantitative growth inhibition assay. Additionally, they showed the same effectiveness as chloramphenicol at 200 µM. On the other hand, **8** and **46** showed antifungal activity towards *Saccharomyces cerevisiae* at 2 mM and 200 µM, respectively. However, **31** exerted its antibacterial activity at 200 µM [50]. Chaetovirdin A (**31**) possessed weak inhibitory potential towards induction of chlamydospore-like cells of the plant pathogen *Cochliobolus lunatus* (40–50% at 100 µg/disc), and prohibition of the rice-blast fungus *Pyricuralia oryzae’s* growth (IC_50_ 2.5 µg/mL) [27]. The new metabolites, 5′-epichaetoviridin A (**33**), 4′-epichaetoviridin F (**48**), 12β-hydroxychaetoviridin C (**41**), and chaetoviridins G-I (**49**, **51**, and **52**), along with chaetoviridins A–E (**31**,**37**, **40**, **43**, and **44**) and 4′-epichaetoviridin A (**32**) separated from the endophytic fungus *C. globosum*, were assessed in an in vivo pathogenicity assay that involved the infection of *Caenorhabditis elegans* with *Enterococcus faecalis* (Figure 8). None of them possessed a significant ability to promote nematode survival [36]. Park et al. stated that chaetoviridins A (**31**) and B (**37**) also possessed growth inhibitory activity against *Magnaporthe grisea* (rice blast) and *Pythium ultimum* (wheat leaf rust) mycelia (MICs 1.23 and 33.3 and 1.23 and 33.3 μg/mL, respectively) in vitro [61]. They also exhibited strong in vivo antifungal activity against *M. grisea* and *Puccnicia recondite* (wheat leaf rust) [36]. Chaetoviridin A (Conc. 62.5 µg/mL) inhibited rice blast development by >80%, but it had moderate control (50%) of tomato late blight at 125 µg/mL. Therefore, they can control wheat leaf rust rice blast and tomato late blight [36,61]. Chaetoviridin A (**31**), chaetoviridin E (**44**), and chaetomugilin D (**8**) separated from a deep sea-derived *Chaetomium* sp. NA-S01-R1 had weak to moderate antibacterial effectiveness against *Vibrio* strains (*V. vulnificus*, *V. rotiferianus*, and *V. campbellii*) (MIC 15.4–32.3 µg/mL) and MRSA (MICs 15.2–32.4 µg/mL), compared to erythromycin (MIC 2.0–7.7 µg/mL) and chloramphenicol (MIC 7.5–7.6 µg/mL) [25].

Chaetoviridins B (**38**) and E (**44**) displayed antibacterial activity towards *E. faecalis* and *S. aureus* [74]. Chaetoviridins A (**31**) and B (**34**) also had antimicrobial activity against *B. subtilis*, *Rhizoctonia solani*, and *E. coli* (IZDs 15 and 14 mm, respectively, towards all strains) [63].

Yan et al. reported that chaetoviridin A (**31**) exhibited significant antifungal potential (EC_50_ 1.97 µg/mL) towards *Sclerotinia sclerotiorum*, which causes rape *Sclerotinia* rot (RSR). Further, **31** displayed in vivo protective efficacy (64.3%, dose 200 µg/mL) towards RSR, comparable to that of carbendazim (69.2%). Additionally, it had antifungal activity towards *Botrytis cinerea*, *Phytophthora capsici*, *Fusarium graminearum*, and *F. moniliforme* (inhibition rates 69.1, 60.7, 77.0, and 59.2%, respectively) [73].

*C. globosum* CEF-082, isolated from cotton plants, produced chaetoviridin A (**31**), which possesses significant antifungal activity towards *Verticillium dahlia*, which causes cotton *Verticillium* wilt (CVW). It induced mycelial deformation and cell necrosis, increased NO and ROS production, prohibited the germination of microsclerotia of *V. dahliae*, and boosted the cotton defensive response [69].

Chaetoviridin A (**31**), 5′-epichaetoviridin A (**33**), and chaetoviridin E (**44**) were separated from the soil-associated *C. globosum* 22–10. They showed significant inhibitory effectiveness (inhibition 32.31%, 15.38%, and 13.85%, respectively) at 100 µg/mL towards *Bipolaris sorokiniana*, a soil-borne pathogen that commonly causes wheat root rot. It is noteworthy that chaetoviridin A had the same inhibitory efficiency as the carbendazim (3 mg/mL), suggesting its potential to be a biocontrol agent for *B. sorokiniana* [67]. Further, chaetoviridin A identified from an EtOAc extract of *C. globosum* F211_UMNG isolated from *P. heptaphyllum* was active towards *F. oxysporum* [66]. Chaetomugilin D (**8**) and chaetoviridin A (**31**) (200 µM) significantly reduced the growth of *B. subtilis* and *Psuedomonas putida* [55].

### 3.3. Phytotoxic Activity

The EtOAc extract of *C. globosum* associated with *Amaranthus viridis* yielded chaetomugilin D (**8**) and chaetomugilin J (**18**), which exhibited phytotoxic potential against lettuce (*Lactuca sativa*) seed germination with IC_50_ 24.2 and 22.6 ppm, respectively, for root growth inhibition, and IC_50_ 27.8 and 21.9 ppm, respectively, for shoot growth inhibition. The results revealed the potential of these metabolites as herbicides or weedicides that can replace hazardous synthetic compounds [51]. Chaetomugilin A (**2**), D (**8**), S (**28**), I (**16**), J (**18**), Q (**25**), and O (**23**) isolated from *C. globosum* TY1 exhibited allelopathic activity towards *Brassica campestris*, *Cucumis sativus*, *Eruca sativa*, *Daucus carota*, *Lactuca sativa*, *Scrophularia ningpoensis*, *Brassica rapa*, and *Spinacia oleracea.* Among them, **23** exhibited higher germination and root and shoot elongation inhibitory potential with lower IC_50_ values and higher response indexes than glyphosate (positive control). Moreover, **2**, **8**, and **28** exhibited similar or better inhibitory effects than glyphosate. At the same time, **2**, **8**, and **23** were more powerful growth inhibitors than **16**, **18**, and **25**, which could be attributed to the existence of a tetrahydrofuran moiety. On the other hand, **23** had a higher growth-suppression effect than those of **2**, **8**, and **28**, suggesting that the lactone rings may reduce the inhibitory effects [45].

### 3.4. Antimalarial and Antimycobacterial Activities

Phonkerd et al. isolated new derivatives, chaetoviridins E and F (**44** and **47**) and 5′-epi-chaetoviridin A (**33**), together with chaetoviridin A (**31**) from *C cochliodes* VTh01 and *C. cochliodes* CTh05. Compound **44** showed antimalarial activity against *P. falciparum* (IC_50_ 2.9 µg/mL)—and was compared to artemisinin (IC_50_ 0.001 µg/mL)—using the microculture radioisotope technique [60]. Additionally, **44** and **47** displayed weak antimycobacterial potential towards *M. tuberculosis* (MIC 50 and 100 µg/mL, respectively), in comparison to isoniazid and kanamycin sulfate in the microplate Alamar Blue assay (MABA) [60].

### 3.5. Anti-Inflammatory Activity

Chaetomugilin D (**8**) from *C. globosum* isolated from damp building materials notably increased TNF-α production in RAW 264.7 murine macrophages. Therefore, **8** contributes to the non-allergy-linked respiratory disorders such as non-allergic asthma and rhinitis for people working and living in damp buildings [52]. Two new derivatives, chaetoviridins J (**53**) and K (**54**), along with 11-*epi*-chaetomugilin I (**17**) and chaetomugilins E (**11**), F (**13**), I (**16**), J (**18**), and N (**22**) biosynthesized by *C. globosum* were isolated from *Wikstroemia uva-ursi* leaves. Their structures were verified by NMR, Mosher’s method, X-ray diffraction, and CD. Chaetoviridin K (**54**) was separated as a mixture of diastereoisomers that could not be purified by chiral columns. Their potential to inhibit TNF-α-induced NF-κB and NO (nitric oxide) production in the LPS-stimulated RAW 264.7 cells was assessed. Compounds **16** and **17** remarkably suppressed TNF-α-induced NF-κB activity (IC_50_ 0.9 µM, at 50 µM), in comparison to BAY-11 (IC_50_ 2.0 µM) and TPCK (IC_50_ 3.8 µM), whereas **11**, **13**, and **18** possessed moderate inhibitory activity (IC_50_ ranging from 5.1 to 11.6 µM, conc. 50 µM). In addition, **11**, **13**, **16**–**18**, and **53** strongly prohibited NO production (74.2–99.9%). It is noteworthy that **16** and **17** powerfully suppressed NO release (IC_50_ 0.3 and 0.8 µM, respectively) more than N-monomethyl-L-arginine (IC_50_ 25.1 µM). On the other hand, **11**, **13**, and **18** also considerably inhibited NO production (IC_50_ values ranging from 1.9 to 5.8 µM); however, **54** exhibited a weak effect [56].

### 3.6. Antidiabetic, Antioxidant, and Antiviral Activities

Chaetomugilins A (**2**), I (**16**), J (**18**), and Q (**25**) and chaetoviridins B (**38**) and J (**53**) purified from the EtOAc extract of *C. globosum* TY-1 isolated from *Ginkgo biloba* bark were tested for α-amylase and α-glucosidase inhibitory activity. Chaetoviridin B (**38**) had promising α-glucosidase inhibitory potential (IC_50_ 6.328 µM), compared to acarbose (IC_50_ 54.7 µM). The other compounds displayed no α-amylase or α-glucosidase inhibitory activity (IC_50_ > 50 µM) compared to acarbose (IC_50_ 13.7 and 54.7 µM, respectively) [47]. Chaetoviridin B (**38**) and chaetomugilin A (**2**) have the same skeleton except for the **2** having one more hydroxyl group in the side chain, revealing that the group is detrimental for α-glucosidase inhibition [47]. Chaetoviridins A (**31**) and B (**37**) purified from an EtOAc extract of *C. globosum* exhibited noticeable antioxidant potential on TLC using DPPH [63]. Additionally, chetomugilin D (**8**) and its analog epi-chetomugilin D (**10**) possessed antiviral activity towards HSV-2 (inhibition 33.3% and 40.7%, respectively, at 25µg/mL [49].

### 3.7. Caspase-3 and Monoamine Oxidase (MAO) Inhibitory Activities

Caspase-3 (cysteine aspartyl-specific protease-3) is one of the executioners in caspase-linked apoptosis that is activated in nearly every apoptosis model [77]. It is a prominent therapeutic target for excessive apoptosis-associated disorders, such as ischemic damage and neurodegenerative disorders (e.g., Huntington’s and Alzheimer’s diseases) and autoimmune disorders [78]. New azaphilones, chaetomugilin S (**27**), 7,5′-bis-epi-chaetoviridin A (**34**), and 7-epi-chaetoviridin E (**45**), purified from the lichen-associated *C. elatum* 89-1-3-1, were isolated from *Ramalina calicaris*. Their absolute configurations were assigned by CD experiments and X-ray crystallography. They exhibited caspase-3 inhibitory potential (IC_50_ 20.6, 10.9, and 7.9 µM, respectively) in the cysteine aspartyl-specific protease-3 enzymatic assay compared with Ac-DEVD-CHO (IC_50_ 13.7 nM) [31]. On the other hand, 31 possessed weak MAO inhibitory potential (IC50 1.2 × 10^−2^ g/mL) [27].

### 3.8. Cholesteryl Ester Transfer Protein Inhibitory Activity

CETP allows the transfer and exchange of neutral lipids such as CE (cholesteryl ester) and TG (triacylglycerol) between plasma and lipoproteins. It is proven to play important role in atherosclerosis [79]. Tomoda et al. reported that chaetoviridin B (**37**) showed CETP (cholesteryl ester transfer protein) inhibitory activity with an IC_50_ < 6.3 µM, whereas chaetoviridin A (**31**) had moderate inhibitory activity (IC_50_ 31.6 µM) [80]. It was indicated that the existence of an electrophilic enone(s) and/or ketone(s) at both C-8 and C-6 of isochromane core is substantial for eliciting activity [80].

### 3.9. Anti-SARS-CoV-2 Activity

The COVID-19 pandemic has affected global health since 2019. COVID-19 can lead to acute respiratory distress syndrome [81]. It is produced by a novel type of coronavirus (CoV) called SARS-CoV-2 that was first found in Wuhan City, China, and then spread worldwide [82,83]. It is considered a highly pathogenic CoV in the human population. The SARS-CoV-2 genome encodes two polyproteins which are processed by a 3C-like protease (3CLpro) and a papain-like protease [84]. 3CLpro (3C-like protease) and PLpro (papain-like protease) are needed for processing the polyproteins into mature nonstructural proteins, such as helicase and RdRp (RNA-dependent RNA polymerase), which are substantial for viral replication and transcription [85]. 3CLpro has high substrate specificity and is also referred to as Mpro (main protease) [86]. 3CLpro’s substrate specificity makes this enzyme an ideal target for developing broad-spectrum antiviral agents [87]. Its inhibitors are expected to have selective toxicity towards the virus [88].

Fungi are a treasure that can provide a remarkable pool of secondary metabolites with antiviral activities [89]. The characterization and discovery of antiviral fungal metabolites is an emerging and promising research field. Recently, many reports have been published on the structure-based virtual screening approach for the repurposing of natural metabolites, hoping to accelerate and assist in the discovery of agents for COVID-19 treatment [82]. We carried out a computational study on the reported fungal chaetomugilins and chaetoviridins was carried out to identify their 3CLpro inhibitory potential, using docking calculations and MD simulations (Table 3, Table 4, Table 5, Table 6 and Table 7). Three crystal structures containing non-covalent inhibitors for the protease (PDB entry: 6W81, 6M2N, and 7K0F) were selected. All the listed metabolites were docked with extra precision for maximum accuracy. The docking method was validated by redocking the inhibitors that co-crystallized with 6W81, 6M2N, and 7K0F; and RMSD values were within an acceptable range and less than 1.50 Å. All the redocked inhibitors revealed the same binding interaction with the active site in the original pose. Further, in silico ADMET (drug absorption, distribution, metabolism, excretion, and toxicity) predictions of the properties of the investigated compounds were carried out. Finally, a molecular dynamics simulation was conducted to evaluate the nature of the ligand–target interaction under simulated physiological conditions for the most compatible drug-like molecule that could be used in pursuit of a truly adequate medication for COVID-19.

#### 3.9.1. Preparations of Ligands and Proteins

**Figure 9:**
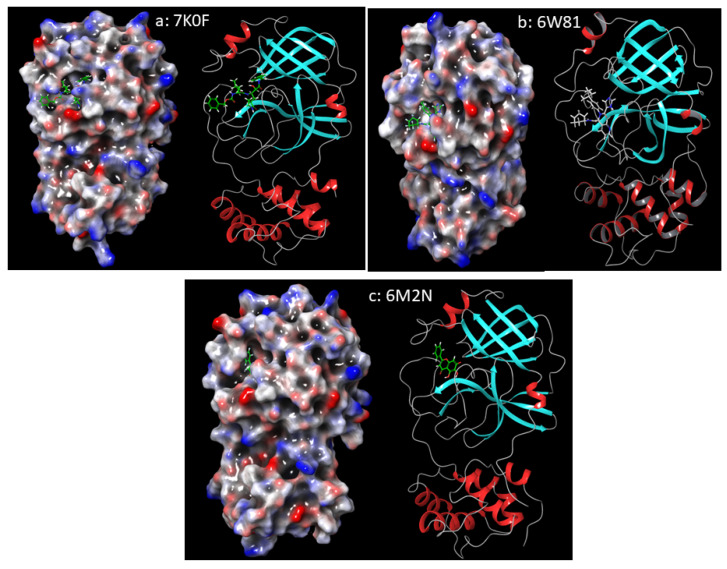
**The** 3CL pro-hydrolase prepared via Protein Preparation Wizard, Maestro. H-bonding network optimized and geometry minimized structures of 2019-nCOV 3CL hydrolase (Mpro)—PDB ID: (**a**) 7K0F, (**b**) 6W81, (**c**), and 6M2N. Represented as 3D molecular surfaces and ribbon structures.

By using *LigPrep*, the conversion of 2D structures to 3D, tautomerization, and ionization yielded 254 minimized 3D structures. The minimized 3D structures were used for docking with the crystal structure of the 3CL hydrolase (Mpro). Preparation of the viral protease (6M2N, 6W81, and 7K0f) by the Protein Preparation Wizard tool optimized the H-bonding network and minimized the geometry. Assurance of assigning the proper formal charges and force field treatments was achieved by adding missing hydrogens and correct ionization states (Figure 9).

#### 3.9.2. Molecular Docking Studies

After defining the grid box in the prepared viral protease via the Receptor Grid Generation tool of Glide in Maestro, the prepared 3D molecular structures were docked into the co-crystallized inhibitor binding site of the viral protease. Table 3 shows the results of the top-score docked ligands chosen based on the most negative docking scores. These scores represent the best bound ligand conformations and relative binding affinities. Chaetovirdin D (**43**) exhibited the highly negative docking scores of −7.944, −8.141, and −6.615 kcal/mol, complexed with 6M2N, 6W81, and 7K0f, respectively. The reference inhibitor exhibited the following scores: −5.377, −6.995, and −8.159 kcal/mol, complexed with 6M2N, 6W81, and 7K0f, respectively.

The docking analysis was updated by re-docking the selected chaetomugilins and chaetoviridins with the three crystal structures of the 3CL pro-hydrolase (PDB ID: 6M2N, 6W81, and 7K0f) at variable resolutions, 2.20, 1.55, and 1.65A, respectively. Analysis of the docking scores of these compounds with the inhibitor binding sites of 2019-nCoV main protease (PDB ID: 6M2N, 6W81, and 7K0f) revealed different docking scores, and hence binding affinities (Table 4, Table 5 and Table 6). These differences can be attributed to the differences in the grid formation due to the presence of different inhibitors in the binding sites of the three crystal structures.

**Figure 10:**
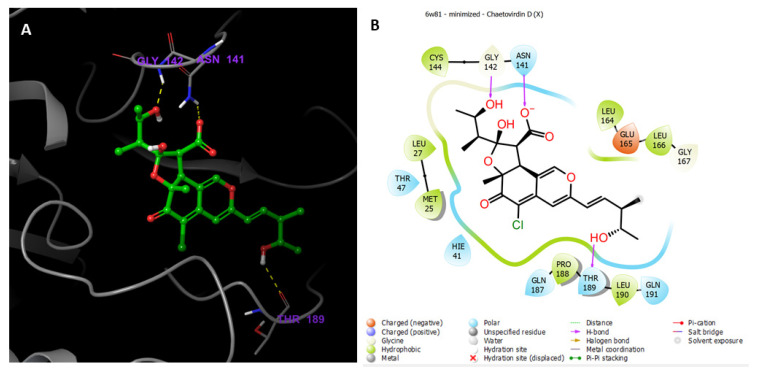
(**A**) Putative binding mode of chaetovirdin D in the binding site of 2019-nCOV 3CL hydrolase (Mpro), PDB: 6W81. Chaetovirdin D is displayed as green sticks. The amino acids of the binding site are represented as grey sticks, and H-bonds are represented as yellow dotted lines. (**B**) 2D depiction of the ligand–protein interactions.

**Figure 11:**
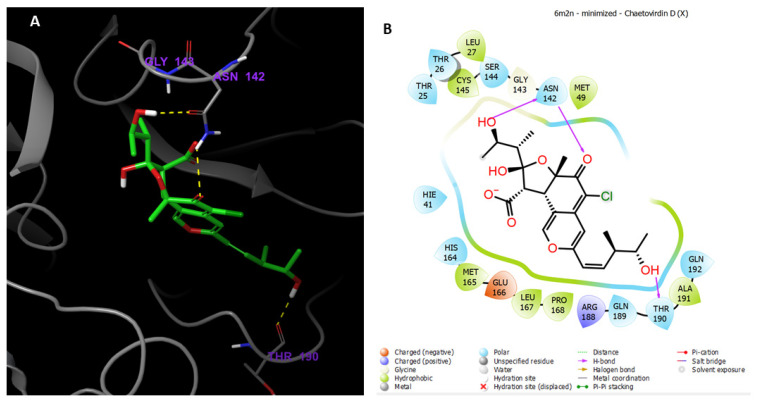
(**A**) Putative binding mode of Chaetovirdin D in the binding site of 2019-nCOV 3CL hydrolase (Mpro), PDB: 6M2N. Chaetovirdin D is displayed as green sticks. The amino acids of the binding site are represented as grey sticks, and H-bonds are represented as yellow dotted lines. (**B**) 2D depiction of the ligand–protein interactions.

**Figure 12:**
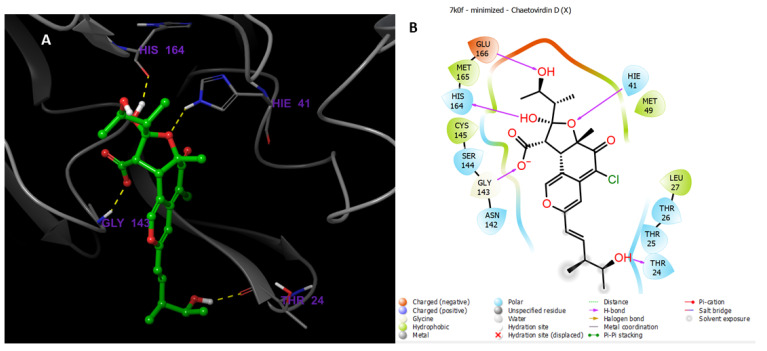
(**A**) Putative binding mode of Chaetovirdin D in the binding site of 2019-nCOV 3CL hydrolase (Mpro), PDB: 7K0F. Chaetovirdin D is displayed as green sticks. The amino acids of the binding site are represented as grey sticks, and H-bonds are represented as yellow dotted lines. (**B**) 2D depiction of the ligand–protein interactions.

Analysis of the docking of **43** revealed that it interacted through hydrogen bonds (Figure 10) with the binding site residues of SARS-CoV-2 main protease (6W81). The binding site residues Asn141, Gly142, and Thr189 of the viral protease exhibited hydrogen bonding with the various hydroxyl groups of **43**. Additionally, **43** interacted through hydrogen bonds (Figure 11) with the binding site residues of SARS-CoV-2 main protease (6M2N). The binding site residues Asn142 and Thr190 of the viral protease showed hydrogen bonding with the various hydroxyl groups of **43**. Its interactions through hydrogen bonds with the binding site residues of SARS-CoV-2 main protease (7K0F) are shown in Figure 12. The binding site residues Thr24, His41, Gly143, His 164, and Glu166 of the viral protease displayed H-bonding with the various hydroxyl groups of **43**.

#### 3.9.3. In Silico ADMET Properties of Selected Ligands

Fifty-three compounds from among the reported fungal chaetomugilins and chaetoviridins were selected and processed using LigPrep of the Schrodinger suite (Schrödinger Release 2021-4: LigPrep, Schrödinger, LLC, New York, NY, USA, 2021; making 3D models with ionization states at pH 7.0 ± 0.2 generated by the OPLS3 force field. The QikProp module of the Schrodinger suite (Schrödinger Release 2021-4: QikProp, Schrödinger, LLC, New York, NY, USA, 2021; was used to predict the ADME properties. The predicted ADMET properties are summarized in Table 7. ADMET analysis describes and determines the biological function, drug-likeness, physicochemical characters, and expected toxicity of compounds. This is meant to evaluate the usefulness of the molecules. The examined descriptors, such as drug-likeness, molecular weight, solvent accessible surface area, dipole moment, hydrogen bond acceptors, donor traits, aqueous solubility, octanol/water coefficient, binding to human serum albumin, number of likely metabolic reactions, brain–blood partition coefficient, human oral absorption, IC_50_ value for blockage of HERG K^+^ channels, central nervous system activity, and number of reactive functional groups, were predicted for the reported metabolites. The values obtained for all the compounds are in the recommended ranges.

#### 3.9.4. Molecular Dynamics Simulation

The docking studies took a static view for the binding of each molecule in the active site of the protein. A molecular dynamics (MD) simulation computes the atoms movements over time. By using Desmond software, the stability and frequency of the **43** complex with the proteases—PDB ID 6M2N, 6W81, and 7K0f—were studied. Three MD simulations were run for complexes with **43** for 100 ns of simulated time; the complexes’ structures were optimized at pH 7.0 ± 2.0. Complex stability was checked by analysis of the interaction map and the RMSD (root mean square deviation) plot of the ligand and protein.

**Figure 13:**
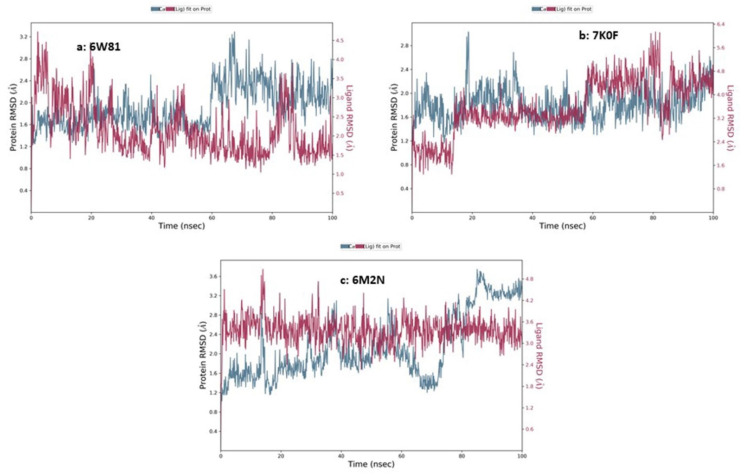
RMSD analysis of MD simulation trajectory. The RMSD plot obtained for (**a**) chaetovirdin D-SARS-CoV-2 main protease complex (PDB ID 6W81), (**b**) chaetovirdin D-SARS-CoV-2 main protease complex (PDB ID 7K0F), and (**c**) chaetovirdin D-SARS-CoV-2 main protease complex (PDB ID 6M2N). The simulated time of 100 ns shows the formation of a stable complex without any significant conformational changes in protein structure.

The RMSD plots in Figure 13a for the chaetovirdin D-SARS-CoV-2 main protease (PDB ID 6W81) complex, Figure 13b for the chaetovirdin D-SARS-CoV-2 main protease (PDB ID 7K0F) complex, and Figure 13c for the chaetovirdin D-SARS-CoV-2 main protease (PDB ID 6M2N) indicate that the complexes tended to stabilize during the simulations (100 ns) with respect to a reference frame at time 0 ns. There were slight fluctuations during the simulations, but within the permissible range of 1–3 Å; hence, they can be considered non-significant. Since the RMSD plots of **43** and protein backbone lie over each other, the formation of a stable complex can be inferred.

**Figure 14:**
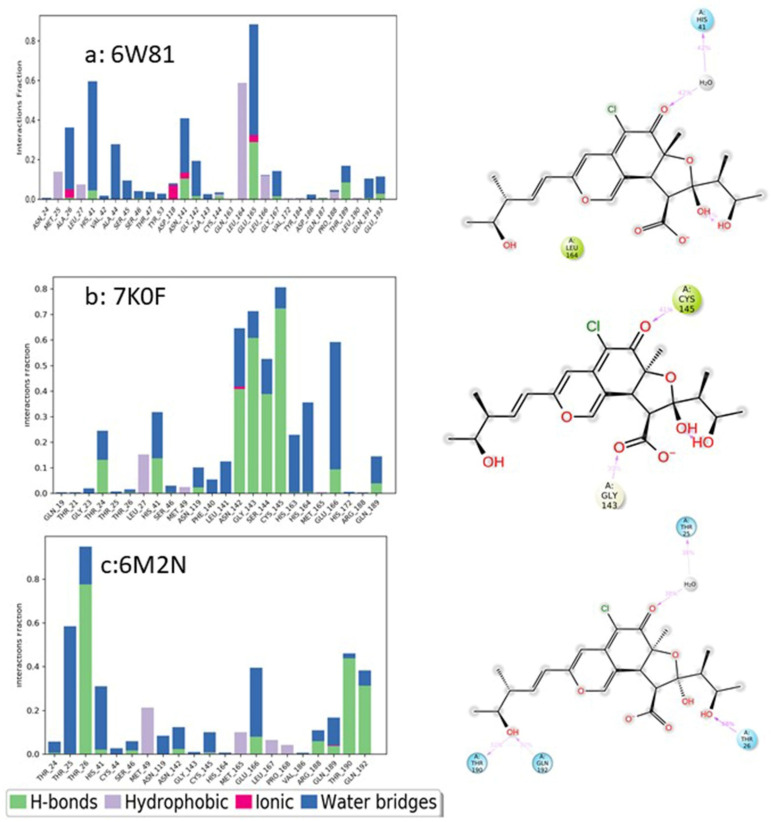
Analysis of molecular interactions and types of contacts with 3CL pro-hydrolase during the MD simulations. Detailed schematic interactions of chaetovirdin D atoms with binding site residues of hydrolase crystal structures (**a**) 6W81, (**b**) 7K0F, and (**c**) 6M2N. Interactions happening during more than 30% of the simulation is shown. Normalized stacked bar charts show the viral protease binding site residues interacting with chaetovirdin D via hydrogen bonds, hydrophobic and ionic interactions, and water bridges.

Figure 14a shows the residue interactions of chaetovirdin D with SARS-CoV-2 main protease (PDB ID 6W81) (the docked poses were retained during the simulation of 100 ns)—i.e., molecular interactions with His41, Asp141, and Glu165 residues. Moreover, a hydrophobic interaction was also established between the Leu165 and aliphatic hydrocarbons of **43**. In Figure 14b, the viral protease (PDB ID 7K0F)-chaetovirdin D contacts over the course of 100 ns are categorized into water bridges, hydrogen bonds, and hydrophobic interactions. The initial docked pose of **43** shows that the important hydrogen bonds (Thr24, His41, Asn142, Gly143, Ser144, Cys145) did not change during the MD simulation. Hydrogen bonding with residue Cys145 was retained for more than 70% of the simulation time. Figure 14c reveals that the viral protease (PDB ID 6M2N)-chaetovirdin D contacts over the course of 100 ns were categorized into water bridges, hydrogen bonds, and hydrophobic interactions. The bar chart shows that hydrogen bonds, hydrophobic contacts, and water bridges prevailed during the course of the simulation. The important hydrogen bonds observed in the initial docked pose of **43** (Thr26, His41, Glu166, Thr190, Gln192) did not change during the MD simulation. Hydrogen bonding with residue Thr26 was retained for more than 5% of the simulation time.

#### 3.9.5. Materials and Methods

##### Preparation of PDB Structures

The three PDB structures (PDB ID: 6M2N, 6W81, and 7K0f) were downloaded from the Protein Data Bank (Protein Data Bank; available online, prepared, and optimized by using “Protein preparation wizard” tool of Schrödinger suite (Schrödinger Release 2021-4: LigPrep, Schrödinger, LLC, New York, NY, USA, 2021) [90]. For this purpose, the bond orders for untemplated residues and known HET groups were assigned and hydrogens were added. Bonds to metals were broken, zero-order bonds between metals and nearby atoms were added, and formal charges to metals and neighboring atoms were corrected. Disulfide bonds were created. Water molecules beyond 5 Å from HET groups were deleted. For ligands, cofactors and metals het states were generated at pH 7.0 ± 2.0 using LigPrep (Schrödinger Release 2021-4: LigPrep, Schrödinger, LLC, New York, NY, USA, 2021). Finally, H-bonds were optimized by using PROPKA [91] at pH 7.0, water molecules beyond 3 Å from HET groups were removed, and restrained minimization was done using the OPLS4 force field.

##### Predictions of ADME Properties 

The ADME properties and drug-likeness of selected compounds were determined in terms of distribution, absorption, metabolism, excretion, etc., via the QikProp module of Maestro Schrodinger (Schrödinger Release 2021-4: QikProp, Schrödinger, LLC, New York, NY, USA, 2021).

##### Receptor Grids Generation and Docking

Glide (Schrödinger Release 2021-4: Glide, Schrödinger, LLC, New York, NY, USA, 2021) was utilized for both grid generation and ligands docking. For docking of fifty-three fungal chaetomugilins and chaetoviridins, three grids were generated using the PDB: 6M2N, 6W81, and 7K0f. For the first grid of PDB 6M2N, the binding region was defined by selecting 3WL. For the second grid PDB 6W81, the binding region was defined by selecting X77. For the third grid PDB 7K0f, the binding region was defined by selecting VR4. The non-polar atoms were set for the VdW radii scaling factor to 1.0, and the partial charge cut-off was 0.25. The ligands docking was performed by using the “ligand docking” tool of Schrödinger suite [92]. The selected protocol was standard precision (SP), the ligand sampling method was flexible, and all the other settings were maintained as default.

##### MD Simulations of Chaetovirdin D in Complexes with 6M2N, 6W81, and 7K0f

MD simulations were run using Schrödinger suite (Schrödinger Release 2021-4: Desmond Molecular Dynamics System, D. E. Shaw Research, New York, NY, USA, 2021. Maestro-Desmond Interoperability Tools, Schrödinger, New York, NY, USA, 2021). The systems of **43** in complexes with 6M2N, 6W81, and 7K0f were retrieved from docking results and first tuned through the “System Builder” tool. The solvent model TIP3P and then orthorhombic shape box shape were selected. The side distances box was set to 10 Å, and the system was neutralized by adding Na^+^ ions. The MD calculations were run for 100 ns per trajectory, with the number of atoms, pressure, and the temperature kept maintained constant (NPT ensemble). Pressure was set to 1.01325 bar and temperature 300.0 K, and the force field was set as OPLS4.

## 4. Biosynthesis of Chaetomugilins and Chaetoviridins

**Scheme 1:**
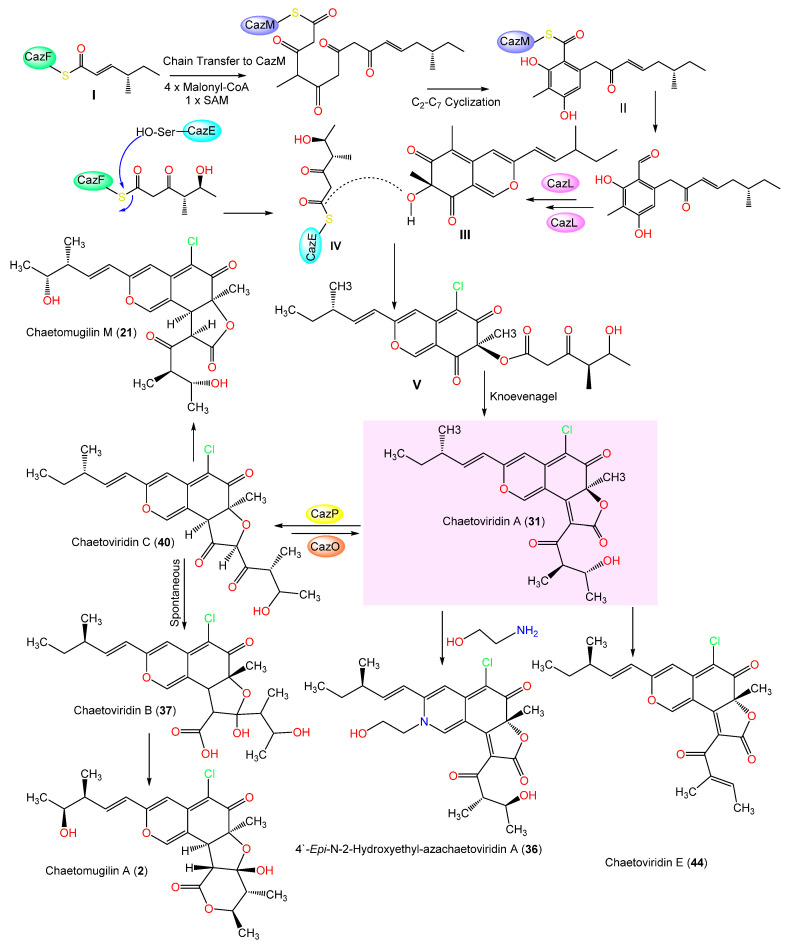
Proposed biosynthesis of chaetomugilins and chaetoviridins [93,94,95,96].

The biosynthetic studies revealed that these metabolites are generated via a polyketide pathway [93,94,95,96]. Their main polyketide chain was produced from malonate and acetate units in a conventional way [93,95]. Briefly, a reduced triketide chain (**I**) was the starting unit to give the aromatic intermediate **II**, which was processed by the halogenase, followed by hydroxylation-stimulated cyclization catalyzed by monooxygenase to yield cazisochromene (**III**, chloropyranoquinone) [96]. Then, the addition of an oxidized triketide unit (**IV**) to **III** by acyltransferase formed the pyranoquinone **V**. The latter underwent a Knoevenagel reaction, resulting in chaetoviridin A (**31**). Chaetoviridin A (**31**) was converted into other chaetomugilins and chaetoviridins through reductions, oxidations, rearrangements, or reactions with amines [93] (Scheme 1).

## 5. Conclusions

**Figure 15:**
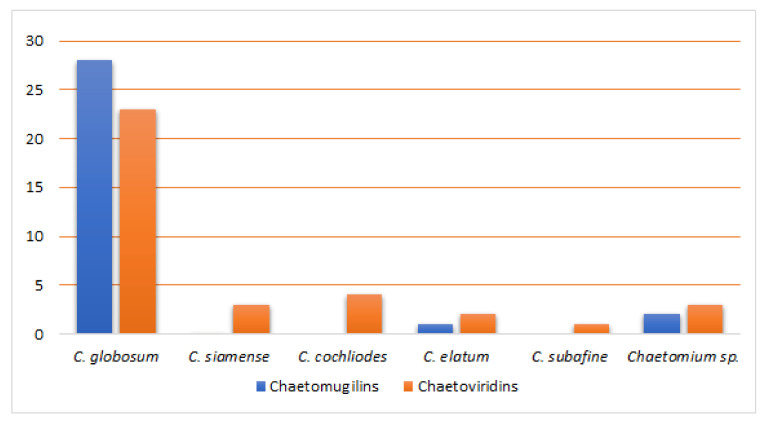
Chaetomugilins and chaetoviridins from various *Chaetomium* species.

**Figure 16:**
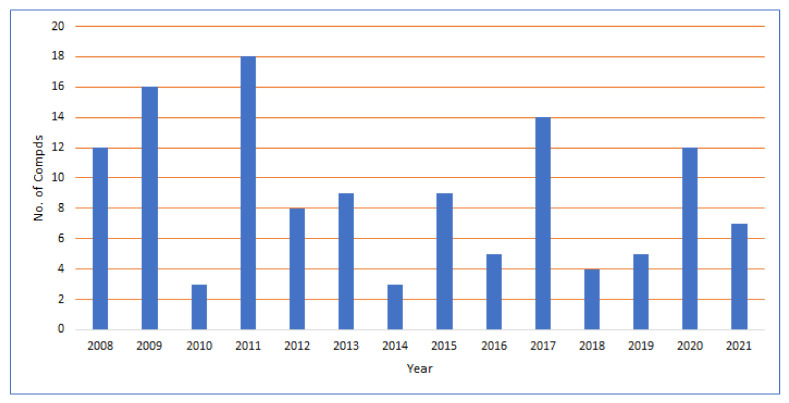
Number of chaetomugilins and chaetoviridins reported per year.

Fungi are important sources of natural polyketide pharmaceuticals with structural complexities that make them interesting and beneficial biometabolites. Chaetomugilins and chaetoviridins are azaphilone derivatives that are mainly sourced from various Chaetomaceae species. In this work, fifty-six metabolites were isolated from four species, *C. globosum*, *C. cochliodes*, *C. siamense*, *C. elatum*, and *C. subafine*, in addition to unidentified *Chaetomium* species. Most of them were from *C. globosum*, as shown in Figure 15. The largest quantities of these metabolites were found in 2011 (18 compounds), 2009 (16 compounds), and 2017 (14 compounds) (Figure 16).

**Figure 17:**
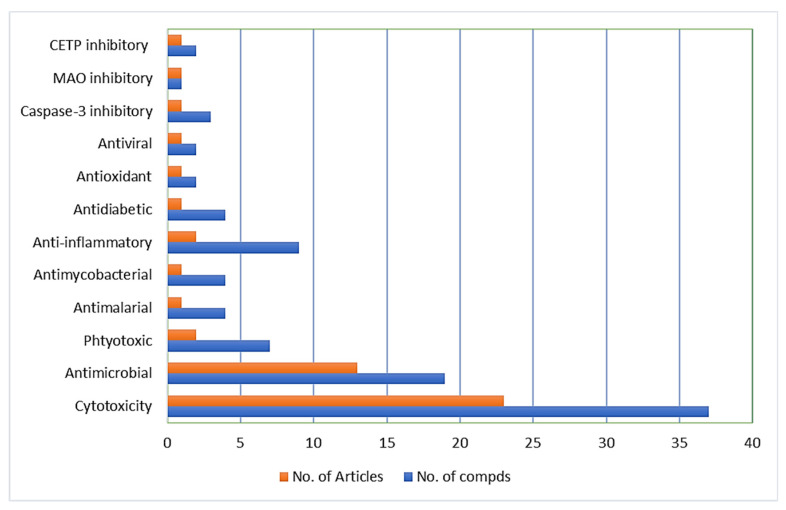
Biological activities of chaetomugilins and chaetoviridins.

These metabolites have been evaluated for diverse bioactivities, such as cytotoxic, antimicrobial, phytotoxic, antimalarial, anti-mycobacterial, anti-inflammatory, antidiabetic, antioxidant, and antiviral ones; and caspase-3, cholesteryl ester transfer protein, and monoamine oxidase (MAO) inhibitory activities (Figure 17). It was found that some chaetomugilins possessed remarkable cytotoxicity towards certain cancer cell lines, equal to or stronger than the effects of control anticancer drugs, such as chaetomugilins C (**7**), F (**13**), I (**16**), and J (**18**).

Some studies revealed that the use of some additional compounds alongside anticancer drugs increased the sensitivity of the cancer cell lines towards these drugs. These metabolites could be further developed into anticancer agents; however, extensive in vivo studies and explorations of their mechanisms of action are needed. Chaetoviridins, particularly chaetoviridin A (**31**), have substantial activity towards different plant pathogens; thus, they might be used as biocontrol agents. Additionally, some reports revealed that chaetomugilins (e.g., A (**2**), D (**8**), O (**23**), and S (**28**)) have serious phytotoxicity, more than the positive controls; therefore, they could be utilized for developing natural eco-friendly herbicides or weedicides that can replace hazardous synthetic compounds. However, extensive studies and field trials should be conducted.

In molecular docking studies, chaetovirdin D exhibited the highly negative docking scores of −7.944, −8.141, and −6.615 kcal/mol, in complexes with 6M2N, 6W81, and 7K0f respectively. The reference inhibitor exhibited the following scores: −5.377, −6.995, and −8.159 kcal/mol, in complexes with 6M2N, 6W81, and 7K0f, respectively. By using molecular dynamics simulations, chaetovirdin D’s stability in complexes with the viral protease was analyzed, and it was found to be stable over the course of 100 ns simulation time.

Undoubtedly, chaetomugilins and chaetoviridins are fungi-derived metabolites that have multiple biological activities. They meet all the requirements for becoming drug leads in their respective therapeutic categories. They have varied chemical compositions that might provide the basis for the synthesis and design of novel and effective pharmaceutical agents. The different substituents at various positions in their skeleton play critical roles in the determination of some of their bioactivities. Finally, studies of the possible mechanisms, biosynthetic pathways, structure–activity relationships, and/or derivatization of these metabolites should be the focus of future research.

## Figures and Tables

**Table 3:** In silico screening results of top candidates of the reported chaetomugilins and chaetoviridins against SARS-CoV-2 3CL protease (PDB: 6W81, 6M2N, and 7K0F).

Name & Structure	For 6W81				For 6M2N				For 7K0F			
	Docking Score	Glide Gscore	Glide Emodel	XP GScore	Docking Score	Glide Gscore	Glide Emodel	XP Gscore	Docking Score	Glide Gscore	Glide Emodel	XP Gscore
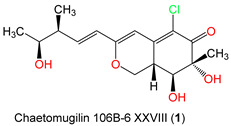	−6.901	−6.918	−45.952	−6.918	−6.862	−6.879	−40.382	−6.879	−6.428	−6.445	−45.402	−6.445
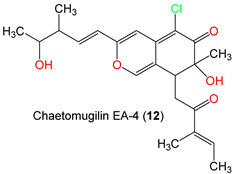	−6.634	−6.933	−53.592	−6.933	−6.657	−7.205	−59.649	−7.205	−6.214	−6.762	−57.163	−6.762
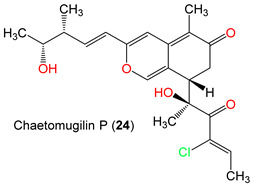	−7.293	−7.294	−52.497	−7.294	−7.851	−7.851	−46.384	−7.851	−6.886	−6.904	−44.124	−6.904
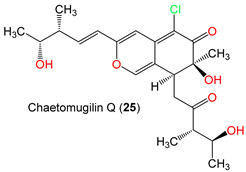	−6.838	−7.269	−59.268	−7.269	−7.4	−7.791	−61.032	−7.791	−6.599	−7.03	−50.901	−7.03
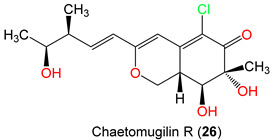	−6.94	−6.958	−40.421	−6.958	−6.921	−6.939	−42.35	−6.939	−6.886	−6.904	−44.124	−6.904
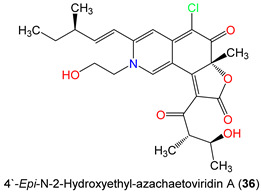	−6.48	−6.48	−57.388	−6.48	−7.519	−7.519	−67.872	−7.519	−7.311	−7.311	−65.197	−7.311
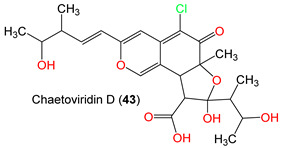	−7.944	−7.944	−51.069	−7.944	−8.141	−8.141	−48.92	−8.141	−6.615	−6.615	−60.199	−6.615
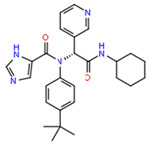 Ref_6W81	−5.377	−5.377	−86.001	−5.377								
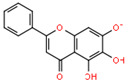 Ref_6M2N					−6.995	−6.995	−52.4	−6.995				
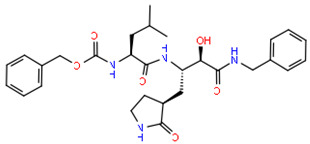 Ref_K0F									−8.159	−8.159	−113.296	−8.159

**Table 4:** In silico screening results of the reported chaetomugilins and chaetoviridins against SARS-CoV-2 3CL protease (PDB: 6W81).

Fungal Chaetomugilins and Chaetoviridins	Docking Score	Glide Gscore	Glide Emodel	XP Gscore
Chaetovirdin D (**43**)	−7.944	−7.944	−51.069	−7.944
Chaetomugilin P (**24**)	−7.293	−7.294	−52.497	−7.294
N-Glutrylchaetovirdin A (**35**)	−7.101	−7.101	−66.739	−7.101
Chaetovirdin B (**37**)	−6.959	−6.959	−37.203	−6.959
Chaetomugilin R (**26**)	−6.94	−6.958	−40.421	−6.958
Chaetomugilin 106B-6 XXVIII (**1**)	−6.901	−6.918	−45.952	−6.918
N-Glutarylchaetovirdin C (**42**)	−6.881	−6.881	−60.87	−6.881
Chaetomugilin Q (**25**)	−6.838	−7.269	−59.268	−7.269
Chaetomugilin EA-4 (**12**)	−6.634	−6.933	−53.592	−6.933
7,4′,5′-*Tris*-*Epi*-chaetovirdin A (**34**)	−6.519	−6.519	−56.68	−6.519
4′-*Epi*-N-2-Hydroxyethyl-azachaetovirdin A (**36**)	−6.48	−6.48	−57.388	−6.48
Chaetomugilin T (**26**)	−6.407	−6.407	−45.87	−6.407
Chaetovirdin J (**53**)	−6.295	−6.673	−54.688	−6.673
12β-Hydroxychaetovirdin C (**41**)	−6.074	−6.08	−54.03	−6.08
Chaetomugilin M (**21**)	−5.985	−6.143	−53.589	−6.143
Chaetovirdin G (**50**)	−5.925	−5.925	−50.419	−5.925
Chaetomugilin I (**16**)	−5.919	−6.467	−53.609	−6.467
N-2-Butyric-azochaetovirdin E (**46**)	−5.862	−5.863	−58.955	−5.863
11-*Epi*-chaetomugilin A (**4**)	−5.771	−5.774	−45.965	−5.774
Chaetovirdin C (**40**)	−5.752	−5.752	−48.221	−5.752
Chaetomugilin H (**15**)	−5.741	−5.741	−40.295	−5.741
N-Glutarylchaetovirdin B (**39**)	−5.733	−5.735	−57.615	−5.735
Chaetomugilin U (**30**)	−5.622	−5.622	−40.195	−5.622
4′-*Epi*-chaetovirdin A (**5**)	−5.561	−5.561	−47.473	−5.561
Chaetomugilin S (**27**)	−5.539	−5.539	−45.867	−5.539
4′,5′-*Bis*-chaetovirdin A (**33**)	−5.507	−5.507	−48.38	−5.507
Chaetovirdin A (**31**)	−5.493	−5.493	−51.732	−5.493
Ref_6W81	−5.377	−5.377	−86.001	−5.377
Chaetovirdin I (**52**)	−5.354	−5.419	−42.206	−5.419
*Seco*-Chaetomugilin D (**9**)	−5.345	−5.347	−45.22	−5.347
Chaetomugilin L (**20**)	−5.25	−5.25	−39.261	−5.25
Chaetomugilin B (**6**)	−5.248	−5.248	−41.211	−5.248
Chaetomugilin A (**2**)	−5.241	−5.243	−44.852	−5.243
11-*Epi*-chaetomugilin I (**17**)	−5.099	−5.647	−54.765	−5.647
Chaetomugilin D (**8**)	−5.093	−5.095	−41.171	−5.095
7-*Epi*-chaetovirdin E (**45**)	−5.089	−5.089	−40.702	−5.089
Chaetovirdin K (**54**)	−4.987	−4.989	−45.166	−4.989
Chaetomugilin N (**22**)	−4.977	−4.977	−3454.68	−4.977
*Seco*-chaetomugilin A (**3**)	−4.926	−4.928	−54.168	−4.928
Chaetomugilin K (**19**)	−4.903	−4.903	−38.494	−4.903
Chaetovirdin H (**51**)	−4.898	−4.898	−51.406	−4.898
4′-*Epi*-chaetovirdin F (**X48**)	−4.873	−4.873	−47.708	−4.873
Chaetovirdin B (**38**)	−4.792	−4.794	−42.458	−4.794
Chaetovirdin E (**44**)	−4.61	−4.61	−48.663	−4.61
Chaetomugilin G (**14**)	−4.516	−4.516	−50.66	−4.516
Chaetomugilin S (**28**)	−4.391	−4.393	−48.292	−4.393
Chaetomugilin F (**13**)	−4.28	−4.28	−40.132	−4.28
Chaetovirdin F (**47**)	−4.271	−4.271	−50.793	−4.271
*Epi*-chaetomugilin D (**10**)	−4.137	−4.139	−36.43	−4.139
Chaetomugilin E (**11**)	−3.597	−3.597	−40.424	−3.597
Chaetomugilin C (**7**)	−3.509	−3.509	−44.435	−3.509
Chaetovirdin G (**49**)	−3.492	−3.492	−48.133	−3.492

**Table 5:** In silico screening results of the reported chaetomugilins and chaetoviridins against SARS-CoV-2 3CL protease (PDB: 6M2N).

Fungal Chaetomugilins and Chaetoviridins	Docking Score	Glide Gscore	Glide Emodel	XP Gscore
Chaetovirdin D (**43**)	−8.141	−8.141	−48.92	−8.141
Chaetovirdin B (**37**)	−7.851	−7.851	−46.384	−7.851
4′-*Epi*-N-2-Hydroxyethyl-azachaetovirdin A (**36**)	−7.519	−7.519	−67.872	−7.519
Chaetomugilin Q (**25**)	−7.4	−7.791	−61.032	−7.791
12β-Hydroxychaetovirdin C (**41**)	−7.045	−7.051	−58.607	−7.051
Ref_6M2N	−6.995	−6.995	−52.4	−6.995
Chaetomugilin R (**26**)	−6.921	−6.939	−42.35	−6.939
Chaetomugilin P (**24**)	−6.9	−6.901	−48.711	−6.901
Chaetomugilin 106B-6 XXVIII (**1**)	−6.862	−6.879	−40.382	−6.879
Chaetomugilin S (**27**)	−6.785	−6.785	−50.138	−6.785
Chaetomugilin EA-4 (**12**)	−6.657	−7.205	−59.649	−7.205
Chaetomugilin M (**21**)	−6.655	−6.813	−56.59	−6.813
7,4′,5′-*Tris*-*Epi*-chaetovirdin A (**34**)	−6.44	−6.44	−55.163	−6.44
Chaetovirdin I (**52**)	−6.439	−6.504	−53.296	−6.504
Chaetovirdin J (**53**)	−6.362	−6.741	−54.219	−6.741
Chaetovirdin G (**50**)	−6.312	−6.312	−48.248	−6.312
Chaetomugilin G (**49**)	−6.272	−6.272	−56.423	−6.272
Chaetovirdin C (**40**)	−6.219	−6.219	−59.734	−6.219
N-Glutarylchaetovirdin B (**39**)	−6.178	−6.179	−53.868	−6.179
11-*Epi*-chaetomugilin I (**17**)	−6.077	−6.625	−61.469	−6.625
Chaetovirdin H (**51**)	−6.071	−6.071	−45.006	−6.071
Chaetovirdin A (**31**)	−5.93	−5.93	−47.164	−5.93
4′,5′-*Bis*-chaetovirdin A (**33**)	−5.896	−5.896	−46.971	−5.896
*Seco*-chaetomugilin A (**3**)	−5.89	−5.892	−44.484	−5.892
Chaetovirdin E (**44**)	−5.871	−5.871	−48.647	−5.871
Chaetomugilin I (**16**)	−5.87	−6.418	−55.891	−6.418
Chaetovirdin F (**47**)	−5.866	−5.866	−54.419	−5.866
4′-*Epi*-chaetovirdin F (**48**)	−5.859	−5.859	−51.775	−5.859
Chaetomugilin F (**13**)	−5.759	−5.759	−43.54	−5.759
N-2-Butyric-azochaetovirdin E (**46**)	−5.453	−5.454	−54.064	−5.454
N-Glutrylchaetovirdin A (**35**)	−5.405	−5.405	−59.359	−5.405
Seco-Chaetomugilin D (**9**)	−5.376	−5.379	−41.076	−5.379
Chaetomugilin H (**15**)	−5.293	−5.293	−52.628	−5.293
Chaetovirdin B (**38**)	−5.217	−5.219	−38.105	−5.219
11-*Epi*-chaetomugilin A (**4**)	−5.166	−5.168	−41.465	−5.168
Chaetovirdin K (**54**)	−5.11	−5.113	−38.902	−5.113
Chaetomugilin A (**2**)	−5.09	−5.092	−36.177	−5.092
Chaetomugilin T (**29**)	−5.012	−5.012	−39.774	−5.012
Chaetomugilin C (**7**)	−4.987	−4.987	−44.839	−4.987
4′-*Epi*-chaetovirdin A (**32**)	−4.905	−4.905	−58.103	−4.905
Chaetomugilin S (**28**)	−4.627	−4.629	−36.966	−4.629
Chaetovirdin G (**49**)	−4.612	−4.612	−42.176	−4.612
7-*Epi*-chaetovirdin E (**45**)	−4.485	−4.485	−51.758	−4.485
*Epi*-chaetomugilin D (**10**)	−4.341	−4.343	−31.912	−4.343
Chaetomugilin U (**30**)	−4.248	−4.249	−40.187	−4.249
Chaetomugilin O (**23**)	−4.208	−4.208	−1767.6	−4.208
Chaetomugilin N (**22**)	−4.07	−4.07	−31.442	−4.07
Chaetomugilin B (**6**)	−4.068	−4.068	−41.99	−4.068
Chaetomugilin K (**19**)	−3.867	−3.867	−34.284	−3.867
Chaetomugilin D (**8**)	−3.667	−3.669	−35.613	−3.669
Chaetomugilin L (**20**)	−3.622	−3.622	−38.371	−3.622
Chaetomugilin E (**11**)	−3.506	−3.506	−41.116	−3.506
N-Glutarylchaetovirdin C (**42**)	−1.412	−1.412	−62.951	−1.412

**Table 6:** In silico screening results of the reported chaetomugilins and chaetoviridins against SARS-CoV-2 3CL protease (PDB: 7K0F).

Fungal Chaetomugilins and Chaetoviridins	Docking Score	Glide Gscore	Glide Emodel	XP Gscore
Ref_7K0F	−8.159	−8.159	−113.296	−8.159
4′-*Epi*-N-2-Hydroxyethyl-azachaetovirdin A (**36**)	−7.311	−7.311	−65.197	−7.311
Chaetomugilin R (**26**)	−6.886	−6.904	−44.124	−6.904
Chaetovirdin D (**43**)	−6.615	−6.615	−60.199	−6.615
Chaetomugilin Q (**25**)	−6.599	−7.03	−50.901	−7.03
Chaetomugilin 106B-6 XXVIII (**1**)	−6.428	−6.445	−45.402	−6.445
Chaetovirdin I (**52**)	−6.381	−6.446	−60.443	−6.446
Chaetomugilin EA-4 (**12**)	−6.214	−6.762	−57.163	−6.762
*Seco*-chaetomugilin A (**3**)	−6.164	−6.166	−54.533	−6.166
Chaetomugilin T (**29**)	−6.003	−6.003	−44.936	−6.003
Chaetomugilin A (**2**)	−5.937	−5.939	−49.214	−5.939
4′-*Epi*-chaetovirdin F (**48**)	−5.781	−5.781	−53.249	−5.781
Chaetovirdin G (**50**)	−5.73	−5.73	−46.915	−5.73
11-*Epi*-chaetomugilin A (**4**)	−5.634	−5.636	−45.502	−5.636
Chaetomugilin M (**21**)	−5.56	−5.718	−41.62	−5.718
Chaetovirdin K (**54**)	−5.521	−5.523	−43.983	−5.523
Chaetovirdin H (**51**)	−5.487	−5.487	−53.211	−5.487
Chaetovirdin J (**53**)	−5.475	−5.853	−56.559	−5.853
Chaetomugilin D (**8**)	−5.457	−5.459	−40.705	−5.459
Chaetomugilin S (**28**)	−5.409	−5.411	−37.945	−5.411
Chaetovirdin A (**31**)	−5.356	−5.356	−55.812	−5.356
Chaetomugilin J (**18**)	−5.262	−5.758	−44.768	−5.758
Chaetovirdin F (**47**)	−5.214	−5.214	−51.016	−5.214
Chaetovirdin E (**44**)	−5.207	−5.207	−57.958	−5.207
Chaetomugilin U (**30**)	−5.17	−5.17	−39.245	−5.17
Chaetomugilin G (**14**)	−5.154	−5.154	−52.872	−5.154
4′,5′-*Bis*-chaetovirdin A (**33**)	−5.147	−5.147	−56.898	−5.147
Chaetomugilin H (**15**)	−5.144	−5.144	−51.211	−5.144
Chaetovirdin C (**40**)	−5.119	−5.119	−51.53	−5.119
*Epi*-chaetomugilin D (**10**)	−5.11	−5.112	−41.394	−5.112
Chaetomugilin P (**24**)	−5.083	−5.085	−47.434	−5.085
Chaetomugilin S (**27**)	−5.005	−5.005	−53.874	−5.005
N-2-Butyric-azochaetovirdin E (**46**)	−4.926	−4.927	−58.939	−4.927
7,4′,5′-*Tris*-*Epi*-chaetovirdin A (**34**)	−4.883	−4.883	−50.65	−4.883
11-*Epi*-chaetomugilin I (**17**)	−4.785	−5.084	−57.348	−5.084
Chaetomugilin B (**6**)	−4.732	−4.732	−44.687	−4.732
Chaetomugilin I (**16**)	−4.534	−4.834	−53.036	−4.834
Chaetomugilin C (**7**)	−4.469	−4.469	−45.833	−4.469
12β-Hydroxychaetovirdin C (**41**)	−4.352	−4.357	−51.515	−4.357
Chaetomugilin E (**11**)	−4.268	−4.268	−39.678	−4.268
N-Glutarylchaetovirdin C (**42**)	−4.247	−4.247	−59.534	−4.247
Chaetomugilin K (**19**)	−4.245	−4.245	−47.953	−4.245
Chaetovirdin G (**59**)	−4.218	−4.218	−46.464	−4.218
*Seco*-Chaetomugilin D (**9**)	−4.205	−4.207	−48.935	−4.207
N-Glutrylchaetovirdin A (**39**)	−4.171	−4.171	−55.598	−4.171
Chaetomugilin F (**13**)	−3.88	−3.88	−42.311	−3.88
Chaetomugilin R (**26**)	−3.808	−5.942	−41.217	−5.942
7-*Epi*-chaetovirdin E (**45**)	−3.699	−3.699	−46.38	−3.699
Chaetomugilin L (**20**)	−3.612	−3.612	−43.218	−3.612
4′-*Epi*-chaetovirdin A (**32**)	−3.552	−3.552	−58.379	−3.552
Chaetomugilin O (**23**)	−2.951	−2.951	31.679	−2.951
Chaetovirdin B (**38**)	−2.642	−2.644	−45.702	−2.644
Chaetomugilin N (**22**)	−0.921	−0.921	−3319.51	−0.921
N-Glutarylchaetovirdin B (**39**)	−0.451	−0.453	−57.257	−0.453

**Table 7:** In silico predicted ADME properties of the selected fungal chaetomugilins and chaetoviridins.

Title	Mol MW	#Stars	Dipole	SASA	Donor HB	accptHB	QPlogPo/w	QPlogS	QPlogKhsa	#Metab	QPlogBB	Percent Human Oral Absorption	QPlogHERG	CNS	#rtvFG
Recommended Range	(130–725)	(0.0–5.0)	(1–12.50)	(300–1000)	(0–6)	(2.0–20.0)	(−2–6.5)	(−6.5–0.5)	(−1.5–1.5)	(1–8)	(−3–1.2)	(<25% Poor; >80% High)	Concern Below −5	(−2 Inactive) (+2 Active)	(0–2)
Chaetovirdin K (**54**)	450.915	0	6.534	733.38	2	7.75	3.725	−6.195	0.507	3	−0.978	100	−5.072	−1	2
Chaetovirdin J (**53**)	408.921	0	9.823	723.174	1	5.95	4.444	−6.019	0.636	6	−1.162	100	−4.971	−2	1
Chaetovirdin I (**52**)	466.914	0	8.017	729.53	3	9.7	2.323	−5.013	0.029	5	−1.839	77.834	−4.801	−2	1
Chaetovirdin H (**51**)	398.455	0	10.648	693.648	0	8.2	2.632	−3.929	−0.18	3	−1.388	87.791	−4.859	−2	0
Chaetovirdin G (**50**)	420.932	0	11.089	718.605	0	6.2	4.554	−5.704	0.494	4	−0.639	100	−4.912	0	1
Chaetovirdin G (**49**)	416.9	0	9.346	698.669	0	4.5	4.914	−6.381	0.89	5	−0.882	100	−4.698	−1	1
4′-*Epi*-chaetovirdin F (**48**)	416.9	0	11.445	723.973	0	7.5	3.722	−5.295	0.155	2	−0.793	100	−5.072	−1	0
Chaetovirdin F (**47**)	416.9	0	11.992	704.483	0	7.5	3.502	−4.926	0.089	2	−0.858	100	−4.864	−1	0
N-2-Butyric-azochaetovirdin E (**46**)	499.99	1	13.922	827.776	1	9.5	4.264	−6.466	0.317	6	−2.11	77.59	−3.438	−2	1
7-*Epi*-chaetovirdin E (**45**)	414.885	0	11.745	707.051	0	7.5	3.482	−5.136	0.123	3	−0.816	100	−4.941	−1	1
Chaetovirdin E (**44**)	414.885	0	11.253	703.096	0	7.5	3.481	−5.061	0.115	3	−0.776	100	−4.877	−1	1
Chaetovirdin D (**43**)	468.93	0	11.587	715.44	3	8.4	3.631	−4.999	0.09	5	−1.572	78.707	−2.816	−2	1
N-Glutarylchaetovirdin C (**42**)	572.053	0	12.347	840.463	0	11.5	3.737	−4.755	0.074	7	−1.845	73.79	−4.594	−2	3
12β-Hydroxychaetovirdin C (**41**)	450.915	0	2.581	723.069	1	6.9	3.938	−5.652	0.514	6	−1.528	92.976	−4.818	−2	0
Chaetovirdin C (**40**)	434.916	1	9.724	758.733	0	5.7	4.847	−6.568	0.718	5	−1.201	100	−5.436	−2	0
Chaetovirdin C (**40**)	434.916	0	8.19	737.905	0	5.7	4.746	−6.172	0.662	5	−1.084	100	−5.134	−2	0
N-Glutarylchaetovirdin B (**39**)	544	0	12.329	793.894	2	11.5	3.491	−5.111	−0.161	7	−2.718	36.417	−0.961	−2	1
Chaetovirdin B (**38**)	434.916	0	5.891	722.451	1	7	4.223	−6.419	0.644	3	−0.608	100	−5.051	0	2
Chaetovirdin B (**37**)	452.931	0	11.095	702.548	2	6.7	4.584	−5.524	0.416	4	−1.112	90.886	−2.679	−2	1
4′-Epi-N-2-Hydroxyethyl-azachaetovirdin A (**36**)	475.968	1	14.774	776.474	1	9.9	3.056	−5.251	0.144	6	−2.061	80.997	−4.863	−2	0
N-Glutrylchaetovirdin A (**35**)	604.095	0	10.792	927.971	0	13.2	3.721	−5.198	−0.147	7	−2.026	76.425	−5.269	−2	2
7,4′,5′-*Tris*-*Epi*-chaetovirdin A (**34**)	432.9	0	12.018	729.829	0	8.2	3.205	−4.877	−0.033	3	−1.26	92.089	−5.078	−2	0
4′,5′-*Bis*-chaetovirdin A (**33**)	432.9	0	10.874	710.174	0	8.2	3.028	−4.504	−0.097	3	−1.245	90.472	−4.825	−2	0
4′-*Epi*-chaetovirdin A (**32**)	432.9	1	10.365	711.04	0	8.2	3.114	−4.52	−0.071	3	−1.186	91.808	−4.763	−2	0
Chaetovirdin A (**31**)	432.9	0	11.776	730.571	0	8.2	3.204	−4.891	−0.033	3	−1.263	92.089	−5.091	−2	0
Chaetomugilin U (**30**)	400.471	0	4.358	680.73	1	7	3.638	−5.427	0.501	3	−0.724	100	−4.736	−1	2
Chaetomugilin T (**29**)	416.47	0	5.838	708.003	2	8.7	2.784	−5.198	0.232	4	−1.243	89.439	−5.03	−2	2
Chaetomugilin S (**28**)	434.916	1	3.205	728.386	1	7	4.315	−6.524	0.659	3	−0.522	100	−5.07	0	2
Chaetomugilin S (**27**)	420.932	0	10.429	713.238	0	6.2	4.401	−5.611	0.469	4	−0.769	100	−4.83	−1	1
Chaetomugilin R (**26**)	328.792	0	6.746	578.157	3	6.9	1.802	−3.7	−0.18	5	−1.075	83.055	−4.136	−2	0
Chaetomugilin Q (**25**)	424.92	0	10.445	738.393	2	7.65	3.474	−5.495	0.295	7	−1.67	90.232	−5.126	−2	1
Chaetomugilin P (**24**)	406.905	0	7.876	701.865	2	6.95	3.532	−5.371	0.361	7	−1.223	95.462	−4.879	−2	1
Chaetomugilin O (**23**)	416.9	1	15.406	699.173	0	5.25	4.493	−6.001	0.677	4	−0.949	100	−4.786	−1	0
Chaetomugilin N (**22**)	432.9	1	14.072	676.058	1	6.95	3.558	−5.135	0.461	5	−1.296	90.388	−4.184	−2	0
Chaetomugilin M (**21**)	450.915	1	12.895	720.323	1	9.9	2.623	−4.609	−0.057	6	−1.517	84.164	−4.712	−2	2
Chaetomugilin L (**20**)	404.933	1	8.629	737.101	0	4	6.159	−7.539	1.184	4	0.149	100	−5.329	1	1
Chaetomugilin K (**19**)	420.932	1	6.053	732.931	1	5.7	5.186	−6.78	0.887	5	−0.327	100	−5.087	0	1
Chaetomugilin J (**18**)	390.906	0	7.403	711.543	1	5.25	4.761	−6.404	0.766	6	−0.712	100	−5.115	−1	1
11-*Epi*-chaetomugilin I (**17**)	406.905	0	8.987	705.067	2	6.95	3.831	−5.476	0.389	7	−0.942	100	−4.938	−1	1
Chaetomugilin I (**16**)	406.905	0	8.14	722.387	2	6.95	3.744	−5.781	0.415	7	−1.23	96.783	−5.14	−2	1
Chaetomugilin H (**15**)	448.942	1	5.803	788.745	1	6.95	4.895	−7.125	0.798	4	−0.859	100	−5.48	−1	0
Chaetomugilin G (**14**)	464.942	0	2.574	796.89	2	8.65	3.897	−6.478	0.455	5	−1.373	96.768	−5.491	−2	0
Chaetomugilin F (**13**)	416.9	0	5.154	721.74	0	6.25	4.481	−6.393	0.6	3	−0.338	100	−5.109	0	0
Chaetomugilin EA-4 (**12**)	406.905	0	7.867	720.199	2	6.95	3.729	−5.743	0.405	7	−1.214	96.92	−5.132	−2	1
Chaetomugilin E (**11**)	448.942	0	6.895	737.896	0	7	4.607	−6.146	0.54	3	−0.279	100	−5.022	0	2
*Epi*-chaetomugilin D (**10**)	434.916	1	5.857	727.72	1	7	4.222	−6.512	0.645	3	−0.622	100	−5.102	0	2
Seco-Chaetomugilin D (**9**)	466.958	1	8.323	773.281	1	6.7	4.99	−6.784	0.844	4	−0.967	100	−5.112	−1	2
Chaetomugilin D (**8**)	434.916	1	5.813	727.059	1	7	4.221	−6.501	0.645	3	−0.621	100	−5.091	0	2
Chaetomugilin C (**7**)	432.9	0	6.592	728.877	1	7.95	3.666	−6.164	0.441	4	−0.828	100	−5.118	−1	0
Chaetomugilin B (**6**)	464.942	0	8.241	745.54	1	8.7	3.831	−6.002	0.415	4	−0.756	100	−5.038	−1	2
11-*Epi*-chaetomugilin A (**4**)	450.915	0	4.613	735.43	2	8.7	3.255	−5.915	0.322	4	−1.091	93.153	−5.126	−2	2
Seco-chaetomugilin A (**3**)	482.957	0	7.988	774.455	2	8.4	3.951	−6.021	0.479	5	−1.427	95.73	−5.066	−2	2
Chaetomugilin A (**2**)	450.915	0	4.464	734.849	2	8.7	3.266	−5.905	0.335	4	−1.106	92.834	−5.084	−2	2
Chaetomugilin 106B-6 XXVIII (**1**)	328.792	0	6.746	578.786	3	6.9	1.79	−3.71	−0.186	5	−1.081	82.981	−4.167	−2	0

#Stars: Number of property or descriptor values that fall outside the 95% range of similar values for known drugs. A large number of stars suggests that a molecule is less drug-like than molecules with few stars. Dipole: Computed dipole moment of the molecule. SASA: Total solvent accessible surface area (SASA) in square angstroms using a probe with a 1.4 Å radius. Donor H-bond: Estimated number of hydrogen bonds that the solute would donate to water molecules in an aqueous solution. Acceptor H-bond: Estimated number of hydrogen bonds that the solute would accept from water molecules in an aqueous solution. QPlogPo/w: Predicted octanol/water partition coefficient. QPlogS: Predicted aqueous solubility, log S. QPlogkhsa: Prediction of binding to human serum albumin. No. of Metabolites: Number of likely metabolic reactions. QplogBB: Predicted brain/blood partition coefficient. % Human Oral Absorption: Predicted human oral absorption on 0 to 100% scale. QPlogHERG: Predicted IC_50_ value for blockage of HERG K^+^ channels. CNS: Predicted central nervous system activity on a –2 (inactive) to +2 (active) scale. #rtvFG: Number of reactive functional groups; the specific groups are listed in the jobname out file. The presence of these groups can lead to false positives in HTS assays and to decomposition, reactivity, or toxicity problems in vivo.

## Data Availability

Not applicable.
